# Functional Analysis of Sporophytic Transcripts Repressed by the Female Gametophyte in the Ovule of *Arabidopsis thaliana*

**DOI:** 10.1371/journal.pone.0076977

**Published:** 2013-10-23

**Authors:** Alma Armenta-Medina, Wilson Huanca-Mamani, Nidia Sanchez-León, Isaac Rodríguez-Arévalo, Jean-Philippe Vielle-Calzada

**Affiliations:** Grupo de Desarrollo Reproductivo y Apomixis, Laboratorio Nacional de Genómica para la Biodiversidad y Departamento de Ingeniería Genética de Plantas, CINVESTAV Irapuato, Irapuato, Mexico; Universidad Miguel Hernández de Elche, Spain

## Abstract

To investigate the genetic and molecular regulation that the female gametophyte could exert over neighboring sporophytic regions of the ovule, we performed a quantitative comparison of global expression in wild-type and *nozzle/sporocyteless (spl)* ovules of *Arabidopsis thaliana* (Arabidopsis), using Massively Parallel Signature Sequencing (MPSS). This comparison resulted in 1517 genes showing at least 3-fold increased expression in ovules lacking a female gametophyte, including those encoding 89 transcription factors, 50 kinases, 25 proteins containing a RNA-recognition motif (RRM), and 20 WD40 repeat proteins. We confirmed that eleven of these genes are either preferentially expressed or exclusive of *spl* ovules lacking a female gametophyte as compared to wild-type, and showed that six are also upregulated in *determinant infertile1 (dif1)*, a meiotic mutant affected in a REC8-like cohesin that is also devoided of female gametophytes. The sporophytic misexpression of *IOREMPTE*, a WD40/transducin repeat gene that is preferentially expressed in the L1 layer of *spl* ovules, caused the arrest of female gametogenesis after differentiation of a functional megaspore. Our results show that in Arabidopsis, the sporophytic-gametophytic cross talk includes a negative regulation of the female gametophyte over specific genes that are detrimental for its growth and development, demonstrating its potential to exert a repressive control over neighboring regions in the ovule.

## Introduction

The ovules of flowering plants are most often formed as elongated primordia emerging from the inner surface of the young carpel, with the integument mounds initiating from periclinal divisions of the epidermal layer [1], [2], [3], [4]. A differentiated ovule is composed of a nucellus and two integuments, and is usually attached by a funiculus to the placental tissue of the gynoecia. The integuments grow to progressively envelop the nucellus; by converging at the apex of the differentiated ovule, they form the micropyle, an extracellular narrow canal through which a pollen tube reaches the female gametophyte to deliver the sperm cells during double fertilization [5]. Through the funiculus, a vascular bundle extends from the placenta to the chalazal region. During early nucellar formation, a single meiocyte undergoes meiosis before giving rise to four haploid products, initiating the gametophytic generation and the formation of the female gametophyte [6]. In *Arabidopsis thaliana*, the megaspore mother cell (MMC) differentiates sub-epidermally, and undergoes meiosis to differentiate a single functional megaspore that divides mitotically to form a female gametophyte composed of the egg cell, two synergids, three antipodals at the chalazal region, and a binucleated central cell whose nuclei fuse prior to fertilization. Following double fertilization, the egg cell and the central cell give rise to the embryo and the endosperm respectively; while the function of synergids is to attract the pollen tube, the function of the antipodals remains unknown.

In Arabidopsis, the investigation of the genetic basis and molecular mechanisms that regulate gametophytic development has recently benefited from large-scale transcriptional analysis and cell-specific isolation methods that allow gene expression comparisons between wild-type and mutant ovules lacking a differentiated female gametophyte, such as *coatlicue* (*coa*), *nozzle/sporocyteless (spl)*, and *determinant infertile1* (*dif1)* [7], [8], [9], [10], [11], Whereas the molecular nature of the gene affected in *coa* remains unknown, *SPL/NZZ* encodes a MADS-like transcriptor factor [12], [13], and *DIF1/SYN1* encodes a meiotic homologue of the *Schizosaccharomyces pombe* REC8/RAD21 cohesin gene [14], [15]. Because distinct mutant phenotypes prevail in the ovules used for each of these experiments, a widely diverse collection of differentially expressed transcripts has been identified, likely due to deregulation of non-equivalent gene collections [11]. Several transcriptomes of distinct cell types of the wild-type female gametophyte are also available [16], [17], [18], allowing direct comparison of gene expression in gametophytic cells and their precursors.

Unlike mature pollen, the differentiated female gametophyte maintains a tight physical contact with the maternal sporophyte that contributes to its protection and nourishment throughout its development [4]. Numerous pleiotropic effects caused by recessive mutations acting at the sporophytic level suggest a cross talk involving genetic and molecular factors that link integumentary and nucellar development to early stages of female gametophyte formation. For instance, Arabidopsis individuals defective in *BELL1*, *AINTEGUMENTA*, *INNER NO OUTER* and *ABERRANT TESTA SHAPE* show a variety of sporophytic defects all detrimental to the formation of the female gametophyte, suggesting a tight control of the sporophyte over the gametophyte [1], [19], [20], [21]. Additional studies in other species showed that the maternal control of sporophytic tissues over the gametic precursor cells initiates early during ovule formation, before MMC differentiation. In rice, the leucine-rich repeat receptor-like kinase *MULTIPLE SPOROCYTE1* (*MSP1*) prevents somatic cells to enter gametogenesis [22], whereas *Oryza sativa TAPETUM DETERMINANT1* (*OsTDL1A*) controls gametic cell specification [23]. On the contrary, no evidence suggests that the female gametophyte could have an influence on the development or physiology of sporophytic cells. Except for the recurrent presence of persistent nucellar cells that are normally reabsorbed by the growing female gametophyte within the inner integumentary region, most gametophytic mutants do not appear to affect the morphology of the differentiated ovule, suggesting a limited influence of gametophytic growth on the sporophyte. Although microarray-based transcriptional profiling resulted in the identification of a few genes that are upregulated in the mutant *coatlicue* [8], it is unclear if the female gametophyte has an influence on the neighboring regions of the ovule.

As a means to identify genes that could be repressed by the female gametophyte in sporophytic tissues of the ovule, we used Massively Parallel Signature Sequencing (MPSS) to identify transcripts that are overrepresented in ovules lacking a female gametophyte as compared to wild-type ovules. We quantified the expression of 1,517 annotated genes that are at least three times more expressed in *spl* as compare to wild-type, in addition to 654 antisense transcripts and 74 expressed signatures corresponding to unannotated intergenic regions. We confirmed that 11 of these genes are either preferentially expressed or exclusive of *spl*, and showed that six of them are also over-expressed in ovules of the meiotic mutant *dif1*, a result suggesting that in wild-type, their ectopic expression is repressed by the female gametophyte. Finally, we show that the misexpression of *IOREMPTE*, a gene encoding a WD40/transducin repeat protein, causes arrest of female gametogenesis and maintenance of the ovule epidermal layer. Our results provide evidence suggesting that the female gametophyte can negatively regulate the sporophytic activity of specific genes that are detrimental for the progression of gametogenesis.

## Materials and Methods

### Plant material and growth conditions

All seeds of *Arabidopsis thaliana* were germinated in Murashige and Skoog (MS) medium under short day conditions (16 hr light/8 hr dark) at 25°C. Seedlings were then transplanted to soil and grown in a greenhouse under long day conditions.

### Statistical analysis of MPSS data

MPSS was performed and analyzed as described in Sanchez-Leon et al. 2012 [11]. All signatures that matched Arabidopsis genomic sequence were analyzed following a previously described classification scheme [24]. The position of each signature was compared with that of genes in the TAIR annotation version 8.0 (www.arabidopsis.org) and assigned to a class based on the position relative to exons and open reading frames [25]. Signatures found in only one MPSS sequencing run across all available libraries or having abundance of less than 4TPM were removed [24]. The MPSS dataset consisted of 13 454 gene tags from *Arabidopsis thaliana* unique loci in the wild-type and *spl* mutant libraries. The total number of gene tags was 1 507 669 for the wild-type and 1 511 244 for *spl*.

The data have been deposited in NCBI's Gene Expression Omnibus [26] and are accessible through GEO Series accession number GSE50020 (http://www.ncbi.nlm.nih.gov/geo/query/acc.cgi?acc=GSE50020). The statistical analysis was performed by the Fisher's exact test for contingency tables [27] as recommended by Auer and Doerge [28], with the modifications described in Sánchez-León et al. (2012) [11].

### RT-PCR and qRT-PCR

Total RNA was isolated from dissected *Landsberg erect*a (L*er*) and *spl* ovules using TRIzol (Invitrogen). Approximately 5 µg of total RNA were treated with 5 U of RNase-free DNase (Boehringer-Mannheim) in 1X DNase buffer (Invitrogen) containing 20 mM MgCl_2_ for 15 minutes (min) at room temperature (RT) and heat inactivated at 65°C for 10 min. RNA was reverse transcribed using 20 pmoles of an oligo(dT) primer (Sigma) in a 50 µl reaction containing 1X RT PCR buffer (Invitrogen), 3 mM MgCl_2_, 0.5 mM of dNTPs, 2.6 mM dithiothreitol, and 200 U of Superscript II reverse transcriptase (Invitrogen). RNA was pre-incubated with the oligo(dT) primer and dNTPs at 65°C for 10 min followed by incubation at 42°C for 2 h; 1 µl of the cDNA samples was used for PCR amplification with 2 mM MgCl_2_, 0.2 mM of each dNTP, 1 U of Taq DNA polymerase (Invitrogen), 13 µL PCR buffer, and 20 pmol of each primer for 30 cycles at an annealing temperature of 60°C. The specific primers used for RT-PCR and qRT-PCR are described in Table S6. Control reactions were carried out using specific primers for the *ACT2* gene. qRT-PCR was performed using the 7500 Real Time PCR System (Applied Biosystems), and using the SYBR Green PCR Master Mix according to the manufacturer's protocol. Gene expression was normalized by subtracting the C_T_ value of the control gene from the C_T_ value of the gene of interest. The Average Expression Ratio (AER) was obtained from the equation AER = 2^−ΔΔCT^, where ΔΔC_T_  =  ΔC_T_ (gene of interest in mutant) – ΔC_T_ (gene of interest in wt), and 2 is the value of the PCR efficiency following the protocol reported by Czechowski et al. (2004) [29].

### Reporter gene fusions and plant transformation

Genomic fragments corresponding to regulatory regions of two genes were amplified by PCR using the primers ProF-At1g47610 (5′-GCTCTAGACAAAATAATGCATTTTGGAAAGC) and ProR-At1g47610 (5′-GGATCCGTCGGAATGCTTGATCC) for At1g47610 and ProF-At2g46680 (5′-AAGCTTGATGTCCCAAAGCATTGAA) and ProR-At2g46680 (5′-GGATCCGATCTGCTCGTCGCTAAACC) for At2g46680, cloned in TOPO8 and later recombined with pMDC162 GATEWAY vector containing the gene uidA gene (GUS) [30]. Genomic fragments included up to 2.5 kb of the regulatory sequence and 500 bp of sequence upstream of the transcriptional initiation codon. The plasmid was introduced into *Agrobacterium tumefaciens* strain GV2260 by electroporation, and *Col* wild-type plants were transformed using the floral dip method [31]. Transformants were selected based on their ability to survive in MS medium with 50 mg hygromicin. Resistant (green seedlings with true leaves) were then transferred onto soil and grown under the conditions described above.

### *In situ* hybridization

The *in situ* hybridization experiments were performed according to the protocol described in Vielle-Calzada et al. (1999) [32]. Fully differentiated gynoecia were fixed in 4% paraformaldehyde and embedded in Paraplast (Fisher Scientific). Samples were cut into 12 µm sections using a microtome and subsequently mounted on ProbeOnPlus slides (Fisher Biotech). A specific probe of 106 bp corresponding to sequence +844 to +950 within the exon of At1g47610 was amplified by PCR and cloned into PCRII-TOPO (Invitrogen). The primers used for amplification were F-At1g47610 (5′-GTGCGGCGGATAAGAAGATA) and R-At1g47610 (5′-ACCACCGCCAAACACTTAAC). The amplified fragment was sequenced to verify transcript orientation. For probe synthesis, the plasmid containing the cloned fragment was linearized with NotI (antisense) and BamHI (sense), and both sense and antisense in vitro transcription was performed using the DIG-labeling kit RNA Labeling Kit (PROMEGA).

### Misexpression of candidate genes

For misexpression experiments in the ovule, the pMDC32 GATEWAY vector [30] was used as it is or modified to replace the *CaMV35S* promoter by either the pNUC1 or *pES1* promoter (Figure S4), without altering its recombination sites. A cDNA of either At1g47610 or At2g46680 was cloned in a pCR8/GW/TOPO vector (Invitrogen) using primers F-At1g47610 (5′-ACTTTCTGAACCTCTTAAGAAACTC) and R-At1g47610 (5′-TTTGACTTGGGTTTTTGTTCT), or F-At2g46680 (5′-ATGCTAAGGAGCCCTCCCAA) and R-At2g46680 (5′-TTTTACATATTAATGCATAACACT). Positive plasmids were recombined with the pMDC32 vectors containing either the CaMV35S, pNUC1, or pES1 promoters following the protocol of Curtis et al 2003. Transformants were generated as described above.

### Whole-mount preparations and histological analysis

Gynoecia from wild-type, mutant or transformant lines were dissected longitudinally with hypodermic needles (1-mL insulin syringes; Becton Dickinson) and fixed with FAA buffer (50% ethanol, 5% acetic acid, and 10% formaldehyde), dehydrated in increasing ethanol concentration, cleared in Herr's solution (phenol:chloral hydrate:85% lactic acid:xylene:oil of clove [1∶1∶1∶0.5∶1]), and observed on a Leica microscope (Wetzlar, Germany) under Nomarski optics. GUS staining assays were conducted as described in Vielle-Calzada et al. (2000) [33].

## Results

### Upregulated genes in *sporocyteless* ovules

Massively Parallel Signature Sequencing (MPSS) is based on methods to clone individual cDNA molecules on microbeads followed by parallel sequencing [34]. MPSS was performed on mRNA isolated from fully differentiated *spl* and wild-type ovules before pollination [11]. After disregarding « non-significant » and « unreliable » signatures [24], [25], sequencing runs were merged and normalized before generating an estimation of their abundance in transcripts per million (TPM). Mapping of all signatures to the Arabidopsis genome resulted in seven classes according to their genomic location. We compared their transcriptional abundance to identify transcripts that were at least three times more abundant (3-fold) in *spl* than in wild-type ovules. Using these criteria, a total of 2,919 signatures were found to be upregulated in *spl* ovules. As expected, the most abundant class corresponds to signatures derived from exon sequences (class 1, representing 52.4% of all signatures; Table 1), followed by signatures that mapped within 500 bp downstream of the STOP codon of a previously annotated coding sequence (class 2, 14.5%). Additionally, 471 sequences correspond to novel antisense transcripts (class 3, 13.7%) and 58 to unannotated intergenic transcribed regions not previously described (class 4, 1.7%). Signatures assigned to class 5 or 7 are less numerous, as they map within introns or splicing sites (0.9 and 1.9%, respectively). Finally, 508 signatures could not be mapped to the genome; unmatched signatures have been previously shown to derive from spliced 3′ ends that not yet have been identified, transcripts found in regions of the genome not yet sequenced, sequencing errors, or non-Arabidopsis RNA contaminants [24].

**Table 1:** MPSS signatures upregulated in *sporocyteless* ovules as compared to wild-type.

CLASS	DESCRIPTION^a^	3 fold expression in *spl* ovules^b^	%	
1	Signature within the exon of the sense predicted ORF	1797	52.4	
2	Signatures within the first 500 bp of 3′ UTR from sense ORF.	497	14.5	
3	Signatures within an exon of antisense ORF	471	13.7	
4	Signatures within intergenic regions	58	1.7	
5	Signatures within introns of sense predicted ORF	31		0.9
6	Signatures within introns of antisense predicted ORF	1		0.03
7	Signatures within exon-intron boundary in sense orientation	64		1.9
0	Signatures with no hit in the genome	508		14.8

a TAIR version 8 (The Arabidopsis Information Resource; www.arabidopsis.org).

b MPSS signatures with at least 4TPM and being expressed at least three times more in *spl* ovules as compared to wild-type ovules.

The sum of class 1, 2, 5 and 7 signatures that mapped to annotated genes allowed the identification and quantification of genes upregulated in *spl* ovules. Of 8770 genes detected at expression levels above 3 TPM in the *spl* library, 1517 were at least three times more expressed in *spl* as compared to wild-type (Table 2 and Table S1). As the expression levels of these genes spread over three orders of magnitude, the most represented class includes genes at transcriptional abundances comprised between 9 and 100 TPM (78.3%). A total of 62 genes are at least 50 times more abundantly expressed in *spl* than in wild-type, with 14 of them showing a fold-change of at least 100 (Table 2).

**Table 2:** Transcriptional distribution of upregulated genes in *sporocyteless* ovules.

	<3TPM		4–8 TPM		9–24- TPM		25–50 TPM		51–100 TPM		101–1000 TPM	>1000 TPM	Total	
**wild-type ovules**	736		250		268		134		76		53	3	1517	
***Sporocyteless*** ** ovules**	0		5		454		449		285		314	10	1517	
**Fold change as compared to wild-type**														
		**3–4**		**5–10**		**11–20**		**21–50**		**51–100**		**>100**	**Total**	
**Genes upregulated in ** ***sporocyteless*** ** ovules**		458		370		359		268		48		14	1517	

All differentially expressed genes were classified according to protein domains available in Pfam, using a cutoff value of E<0.01 [35]. A total of 1335 genes (88%) could be grouped in the 12 different classes shown in Table S2. Whereas genes involved in metabolism and other housekeeping processes are predominant (354 and 286, respectively), 149 encode for signaling proteins, including 50 genes containing a kinase domain, and 30 containing leucine-rich repeat domains. Important genes present in this class include *BRL3*, a gene encoding for a brassinosteroid receptor protein, two members of the *STRUBBELIG*-receptor family (*SRF1*, *SRF7*), the receptor-like kinase gene *ERECTA2* that promotes cell division after integument initiation [36], and *PINOID*, encoding a serine/threonine kinase that acts as a positive regulator of cellular auxin efflux [37], An additional class includes 25 genes encoding proteins with RNA-recognition motifs (RRM) that provide RNA affinity; among them, *Arabidopsis Mei-like3* (*AML3*) was reported as a member of the *mei2*-like genes expressed in the developing embryo, and showing the highest frequency of alternative splicing in Arabidopsis [38], [39], and *RNA-dependent RNA polymerase1* (*RDR1*) participates in silencing pathways that target viruses in several plant species [40]. Other classes include 25 genes encoding peptidases of the insulinase and substilase type, 20 genes encoding WD40 proteins, some of which are involved in the regulation in the response to abscisic acid [41], 20 genes encoding pentatricopeptide-repeat proteins, 16 genes encoding proteins with tetratricopeptide repeats (TPR) motifs, and 15 genes encoding putative helicases.

### Experimental validation of upregulated genes in ovules lacking a female gametophyte

**Figure 1:**
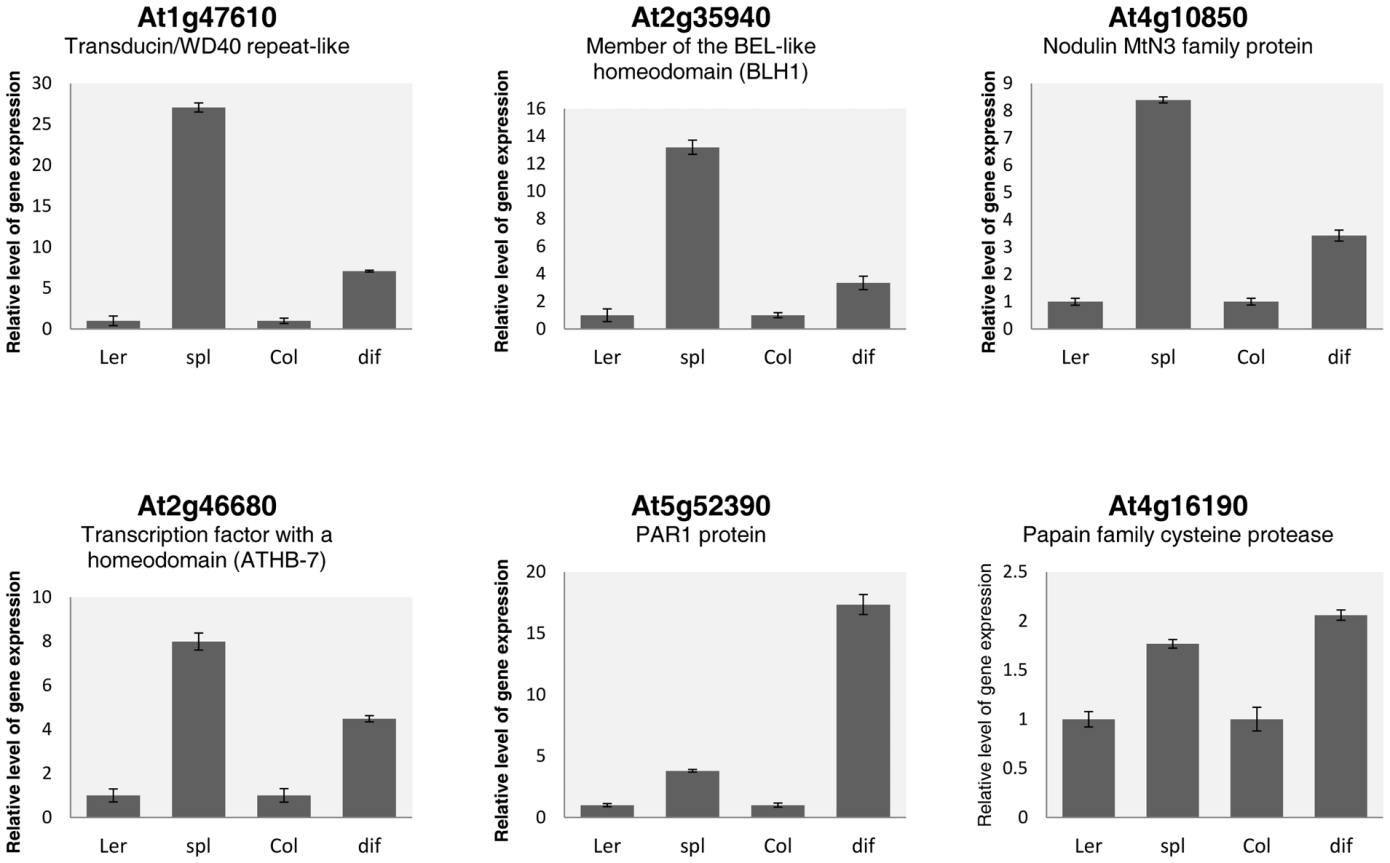
Real-time RT-PCR analysis of upregulated genes *spl* and *dif1* ovules. Expression profiles of At1g47610, At2g35940, At4g10850, At2g46680, At5g52390 and At4g16190 in wild-type, *spl* and *dif1* ovules. The relative level of gene expression of each gene in *spl* ovules was calculated in comparison to *Landsberg* (L*er*) wild-type ovules, while relative level of gene expression of each gene in *dif1* ovules was calculated in comparison to *Columbia* (*Col*) wild-type ovules. Each histogram represents the mean of 3 biological replicates ± standard deviation.

We used both reverse transcriptase PCR (RT-PCR) and real time RT-PCR (qRT-PCR) to confirm that a sample of the MPSS differentially detected genes are upregulated in *spl* ovules. To avoid a possible bias caused by potentially high transcriptional levels [11], we randomly selected a group of eleven genes showing abundances comprised between 13 and 78 TPM in *spl* ovules but not detected in wild-type ovules by MPSS. Conventional RT-PCR on these eleven genes confirmed they were more abundantly expressed in *spl* than in wild-type (Figure S1). Whereas six were expressed in *spl* but not in wild-type ovules, the other five showed preferential expression in *spl*, indicating that some transcripts could be present in wild-type ovules at abundances below the level of detection of our MPSS experiments. We confirmed by qRT-PCR the differential expression of six previously tested genes (Figure 1). At1g47610 encodes a transducin of the WD40 repeat-like superfamily that is 27-fold more expressed in *spl* than wild-type ovules, while At2g35940 encodes BLH1, a *BELL*-like homeodomain protein family that is 13-fold more expressed in *spl* ovules than in wild-type. Interestingly, the misexpression of *BLH1* in the female gametophyte of Arabidopsis results in a cell fate switch of synergid to egg cell [42]. At2g46680 encodes ATHB-7, a transcription factor that contains a homeodomain closely linked to a leucine zipper motif, and shows a 7.9– fold increased expression in *spl* ovules as compared to wild-type; At4g10850 encodes a member of the nodulin MtN3 family and shows 8.3-fold increased expression in *spl* ovules. Finally, At5g52390 encodes PHYTOCHROME RAPIDLY REGULATED1 (PAR1), a bHLH transcription factor that acts as a direct transcriptional repressor of auxin, shows 3.8-fold more expression in *spl* ovules, whereas At4g16190 encodes a cysteine-type peptidase with a 1.7 fold increase expression in *spl* as compared to wild-type. Because abnormal transcriptional activity in *spl* ovules could result from negative regulation by *SPL* activity, we analyzed the expression of the same six differentially expressed genes in mutants of *DIF1*, a strictly meiotic gene that is required for chromosome segregation and belongs to the *REC8/RAD21* cohesin gene family from fission yeast [14]. Homozygous *dif1* individuals are male and female sterile, with multiple chromosome univalents, and defects including meiotic products of uneven size, shape and of variable ploidy. Consequently, *dif1* plants do not initiate megagametogenesis and differentiate ovules lacking a female gametophyte [10]. An ATH1 microarray-based analysis indicates that transcriptional levels of *DIF1* are equivalent in wild-type and *spl* ovules [7], indicating that *DIF1* is not transcriptionally regulated by *SPL* and that these two genes belong to independent regulatory pathways. All six genes also showed significant overexpression in *dif1* ovules as compared to wild-type, with transcriptional abundances ranging between 2 and 17.3 times more expression in *dif1*. These results suggest that the repression of At1g47610, *BLH1*, At4g10850, *ATHB-7*, *PAR1*, and At4g16190 in sporophytic cells of the ovule does not depend on the activity of *SPL per se,* but rather on the development of a female gametophyte within the ovule.

**Figure 2:**
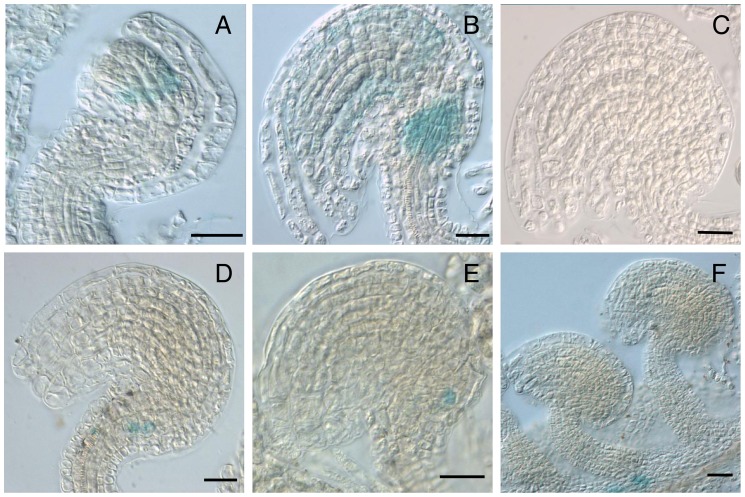
Differential pattern of reporter gene expression in *spl* and wild-type ovules. (**A–C**) *pAt4g16190::GUS* and (**D–F**) *pAt2g46680::GUS.* (**A**) GUS expression in post-meiotic *spl* ovules of *pAt4g16190::GUS* transformants. (**B**) GUS expression of in fully differentiated *spl* ovules of *pAt4g16190::GUS* transformants. (**C**) Absence of GUS expression in fully differentiated *wild-type* ovules of pAt4g16190:GUS transformants (**D**) GUS expression in fully differentiated *spl* ovules of *pAt2g46680::GUS* transformants (**E**) GUS expression in fully differentiated *spl* ovules of *pAt2g46680::GUS* transformants (**F**) Absence of GUS expression in fully differentiated wild-type ovules of *pAt2g46680::GUS* transformants; GUS expression is restricted to a cluster of cells of the placental tissue. Scale bars: 20 μm.

**Figure 3:**
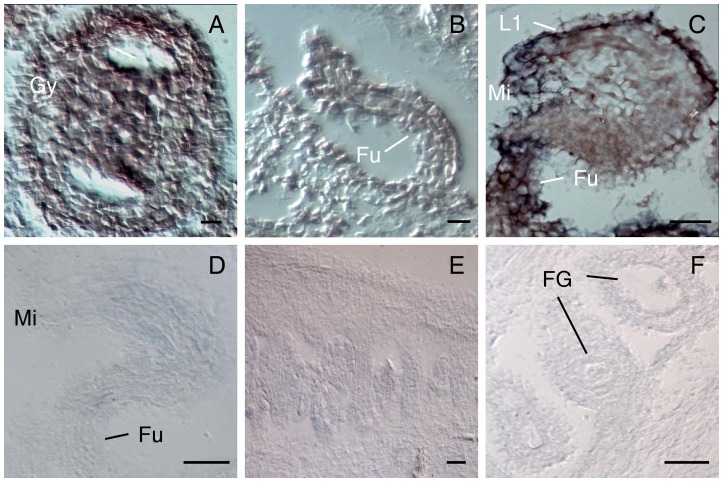
In situ localization of *IOREMPTE* (At1g47610) mRNA in *spl* and wild-type ovules. (**A**) Developing *spl* gynoecia at the onset of ovule formation and hybridized with an antisense *IOREMPTE* probe. (**B**) Developing *spl* ovule at initial stages of integumentary formation hybridized with an antisense *IOREMPTE* probe. (**C**) Fully differentiated *spl* ovule hybridized with an antisense *IOREMPTE* probe. (**D**) Fully differentiated *spl* ovule hybridized with a sense *IOREMPTE* probe. (**E**) Developing wild-type ovules hybridized with an antisense *IOREMPTE* probe. (**F**) Fully differentiated wild-type ovule hybridized with an antisense *IOREMPTE* probe. Abbreviations: FG = female gametophyte; Fu = funiculus; Gy = gynoecium; L1 = L1 layer; Mi = micropyle. Scale bars: 20 μm.

To determine the pattern of expression of some of these candidates, we selected three of the genes that were previously shown to be consistently upregulated in spl and dif1 ovules by qRT-PCR. For At4g16190 and *ATHB-7*, we constructed a *promoter::reporter* fusion cassette by cloning in front of the *uidA* (GUS) gene a genomic fragment that included a putative regulatory region and the initial portion of the coding sequence of the corresponding gene (see Methods for details). To ensure that the observed patterns were reproducible, we examined GUS expression during wild-type ovule development in at least 5 independent transgenic lines for each promoter fusion, and at least 5 individuals for each line (Figure 2). Wild-type transformant plants harboring each of the selected constructs were crossed to *spl/+* and homozygous individuals lacking a female gametophyte were selected for analysis of GUS expression. In wild-type, all transformed lines harboring promoter fusions corresponding to At4g16190 and *ATHB-7* showed GUS expression in seedlings and roots (Figure S2). Fusions corresponding to At4g16190 showed additional expression in the stigma and the endothecium, and those corresponding to *ATHB-7* in the placenta of the gynoecium (Figure S2). Whereas none of the wild-type transformants showed GUS expression in the developing or fully differentiated ovule, fusions corresponding to At4g16190 showed initial reporter expression in the proximal region of the developing *spl* ovule at the end of megasporogenesis (Figure 2). The pattern of expression includes a ring of nucellar and inner integumentary cells that surrounds the region of differentiation of the functional megaspore. At later stages, GUS is expressed in the growing inner integument, but also in a cluster of cells localized in the basal region of the differentiated ovule (Figure 2A and 2B). By contrast, transformant lines corresponding to *ATHB-7* do not show GUS expression at early stages of ovule development. In fully differentiated ovules, GUS expression is restricted to a handful of cells localized in the basal-dorsal region of the ovule, in the vicinity of the funiculus (Figure 2D and 2E). To investigate the pattern of At1g47610 mRNA localization, we conducted *in situ* hybridization in thick sections of *spl* and wild-type ovules, using an antisense probe that corresponds to a 106 bp fragment in 3′ region of its only exon (see Materials and Methods for details). In *spl* ovules before meiosis, At1g47610 mRNA was localized throughout the incipient ovule primordium, including the placental ridge (Figure 3A); however, during megasporogenesis and at subsequent developmental stages, the At1g47610 mRNA was preferentially localized in cells of the L1 (epidermal) layer (Figure 3B and 3C), but also in cells of the outer integument at the micropylar region, and in the funiculus (Figure 3C). By contrast, the At1g47610 mRNA was absent from wild-type ovules throughout development (Figure 3E and 3F). These results show that in the absence of a female gametophyte, MPSS-detected genes are ectopically expressed in distinct and specific regions of the ovule.

### Misexpression of upregulated genes results in reduced fertility

A microarray-based analysis of gene expression of *ATHB-7* reveals that this gene is preferentially expressed in floral organs excluding the gynoecium; in the case of At1g47610, a similar analysis indicates that the gene is expressed in developing seeds at the heart stage of embryogenesis and in mature stamens, but the corresponding transcript is not detected in any additional reproductive or vegetative tissues [43], [44]. We conducted a detailed genetic screen of all segregating insertional lines that are available in public collections for both genes, but did not identify phenotypic defects associated with loss-of-function of any of these two genes, suggesting that their activity could be redundant with other functional elements. To determine if the misexpression of genes overexpressed in mutants lacking a female gametophyte could result in defective ovule development, we generated transgenic lines misexpressing *ATHB-7* or At1g47610 under the control of the cauliflower mosaic virus 35S promoter (*CaMV35S*), or under the control of two specific promoters, namely *pNUC1* and *pES1*. Whereas the *CaMV35S* constitutively drives expression in sporophytic but not gametophytic cells of the ovule [45], [46], *pNUC1* drives expression in the sporophytic but not the gametophytic ovule during megagametogenesis, with strong expression in the funiculus, and *pES1* is active in the developing female gametophyte from the 2-nuclear to the fully cellularized stage [47] (Figure S3). A total of 89 transformant lines were generated (47 for At1g47610, and 42 for *ATHB-7*), including transformants with each of the three selected promoters. This complete collection was scored for fertility defects by quantification of unfertilized ovules and aborted seeds in developing siliques, and detailed observation of whole-mounted cleared ovules in defective plants (Table S3 and Table S4). In the case of misexpression of *ATHB-7*, 11 of 42 transformants showed variable degrees of reduced fertility, with reductions reaching 64.9%. Defective individuals were identified in transformants corresponding to the *CaMV35S*, *pNUC1*, and *pES1* promoters. In the case of At1g47610, 9 out of 47 transformants showed variable degrees of reduced fertility, with reductions ranging between 27.5 and 74.1%. In this case, defective individuals were only identified in transformants corresponding to *CaMV35S* and *pNUC1*, but not to the *pES1* promoter, for which 16 analyzed transformant lines did not show fertility or gametophytic defects at frequencies significantly different from wild-type (results not shown). These results suggest that contrary to misexpression of *ATHB-7* that results in abnormal fertility when expression is driven in both sporophytic and gametophytic cells of the ovule, reduced fertility in transformant lines misexpressing At1g47610 occurs when the gene is expressed in sporophytic but not female gametophytic cells.

### Sporophytic expression of At1g47610 in the ovule results in arrested female gametogenesis

**Figure 4:**
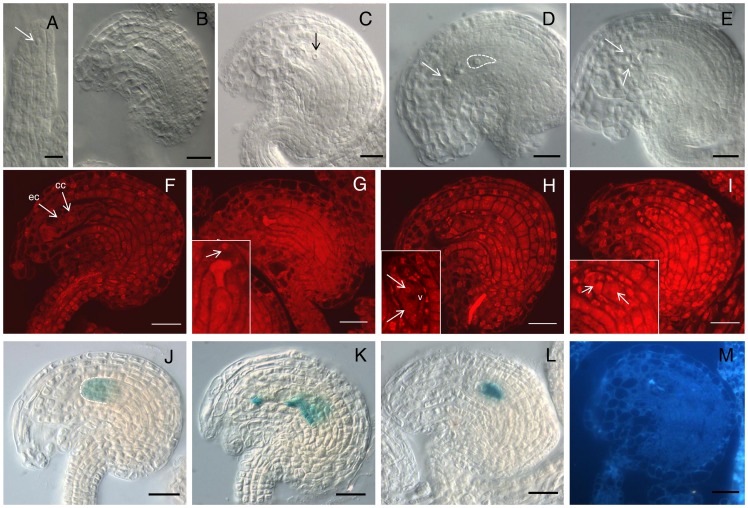
Ovule development in *CaMV35S::IOREMPTE* transformants and wild-type plants. **(A)** to (E) and (J) to (L) correspond to whole-mounted cleared ovules under Nomarsky microscopy; (F) to (I) are whole mounted ovules stained with propidium iodide and observed under confocal microscopy; (M) is a semi-thin (2 um) section observed under bright field. (**A**) Developing ovule of a *CaMV35S::IOREMPTE* transformant at pre-meiotic stage showing normal MMC differentiation. (**B**) and (**C**) Fully differentiated ovules of a *CaMV35S::IOREMPTE* transformant arrested at the functional megaspore stage showing maintenance of the L1 layer and a single gametophytic nucleus (arrow in C). (**D**) and (**E**) Fully differentiated ovules of a *CaMV35S::IOREMPTE* transformant arrested at the functional megaspore stage (dashed line) and showing enlargement of nuclei within prevalent L1 cells (arrows). (**F**) Fully differentiated ovule in wild-type showing the egg cell (ec) and central cell (cc). (**G**) Developing ovule of a *CaMV35S::IOREMPTE* transformant arrested at the functional megaspore stage and showing the prevalence of a cell with enlarged nucleus in the L1 layer (arrow). (**H**) Fully differentiated ovule of a *CaMV35S::IOREMPTE* transformant arrested at the two-nuclear stage, with gametophytic nuclei (arrows) separated by a vacuole (v). (**I**) Fully differentiated ovule of a *CaMV35S::IOREMPTE* transformant arrested at the two-nuclear stage, with gametophytic nuclei (arrows) separated by a cell wall, (**J**) Expression of the *pSPL::GUS* marker in the differentiated ovule of a *CaMV35S::IOREMPTE* transformant showing prevalence of the L1 cell layer. (**K**) Expression of the *pSPL::GUS* marker in the differentiated wild-type ovule showing disruption of the L1 cell layer. (**L**) Expression of *pFM2::GUS* marker in the differentiated ovule of a *CaMV35S::IOREMPTE* transformant arrested at the functional megaspore stage. (**M**) Aniline blue staining to mark degenearated megaspores in the differentiated ovule of a *CaMV35S::IOREMPTE* transformant. Scale bars, A: 10 μm; B-M:20 μm.

To investigate the nature of the fertility defect found in lines misexpressing At1g47610, we conducted a detailed cytological analysis of ovule development in 5 independent transformant lines corresponding to the *CaMV35S* promoter, and compare to ovule formation in the wild-type. Transformant mutant lines misexpressing At1g47610 were named *iorempte* (*ior*), which means ‘to engender’ in native yaqui language. At premeiotic stages, *ior* individuals did not result in phenotypes distinguishable from the wild type. All five lines analyzed showed the same type of defects during ovule development (Table 3 and Figure 4). At the end of meiosis, while most wild-type ovules underwent gametogenesis to form a 2-nuclear female gametogenesis (Figure S4), a large percentage of ovules in all 5 transformants arrested female gametogenesis at the functional megaspore stage of gametogenesis (Figure 4B and 4G), giving rise to fully differentiated ovules that do not form a cellularized female gametophyte, and show a single conspicuous cell with a large nucleus in the prevalent nucellus (Figure 4B to 4G). In some cases, the female gametophyte undergoes a single nuclear division, giving rise to a normally vacuolated 2-nuclear female gametophyte (Figure 4H), or infrequently forming two cells separated by a wall (Figure 4I). In these defective ovules, the L1 layer derived from the young ovule primordium does not degenerate, maintaining large nuclei in cells located at the micropylar pole (Figure 4C, 4E, and 4G). The maintenance of both the integrity in the L1 layer, and the rest of the nucellus, were consistent with the expression of GUS reporter gene under the control of the *SPL* promoter (Figure 4J). The prevalence of a differentiated functional megaspore (FM) in most ovules was confirmed using the *pFM2* molecular marker [48] (Figure 4L). Cellular localization of aniline blue confirmed that meiosis proceeds normally in *ior* individuals (Figure 4M), suggesting that the misexpression of At1g47610 in sporophytic cells of the ovule results in the arrest of gametogenesis at the onset of megagametogenesis.

**Table 3:** Quantitative analysis of defective female gametogenesis in fully differentiated ovules of misexpressing transformants of *IOREMPTE* (*IOR*) and *ATBH-7*.

Transformant line	n	Wild- type	FM stage	Empty^a^	Other^b^
*pNUC::IOR*	663	197(30%)	298(45%)	62(9%)	70(11%)
*CaMV35S::IOR*	290	117(40%)	114(39%)	10(4%)	49(17%)
*pNUC::ATHB-7*	341	72(21%)	243(71%)	12(4%)	14(4%)
C*aMV35S:: ATHB-7*	187	95(51%)	80(41%)	4(2%)	11(6%)
*pES1::ATHB-7*	341	200(59%)	125(37%)	5(1%)	11(3%)

a Includes ovules in which gametophytic nuclei were absent from the nucellus.

b Includes ovules showing female gametophyte at the 2-nuclear stage, female gametophyte with two nuclei separated by a wall, two female gametophyte separated by a wall one at 2n and 4n.

## Discussion

A large body of molecular, genetic and physiological evidence indicates that growth and development of the diploid sporophytic tissues of the ovule are fundamental for female gametogenesis, double fertilization and seed formation [1], [19], [20], [21], [49], [50]. By contrast, only a limited number of studies have addressed the possibility that the female gametophyte could regulate the activity of genes expressed in the surrounding sporophyte. In a previous study, the gametophytic mutant *coatlicue* (*coa*) was used for microarray-based comparisons to identify 527 genes that could be upregulated in ovules lacking a female gametophyte [8]. Here, we took advantage of MPSS-based large-scale transcriptional comparisons to identify 1,517 genes that are upregulated in *spl* ovules lacking female gametophyte as compared to wild-type. Our collection encodes a wide variety of proteins, including a large group of signaling proteins such as receptor-like kinases, RNA binding proteins, and transcription factors, suggesting that the absence of a female gametophyte results in a general deregulation of transcriptional activity in the ovule. When compared to results reported by Johnston et al (2007) [8], 24 genes are shared among the two datasets (Table S5), including *MATERNAL EMBRYO ARREST26* (*MEE26*), At5g62210 encoding a protein expressed in developing seeds following fertilization, *PAR1* (At5g52390) [51], and *IOR* (At1g47610); misexpression of both *PAR1* and *IOR* in ovules lacking a female gametophyte was confirmed by the qRT-PCR in *spl* and *dif1* (Figure 1). In contrast to *in situ* hybridization evidence showing that the upregulated genes in the Johnston et al. dataset are preferentially expressed in the septum and the walls of the gynoecia [8], the expression of At4g16190, *ATHB-7*, and *IOR* tends to be restricted to specific sectors of the ovule, revealing a short distance cross-talk between the female gametophyte and neighboring sporophytic cells.

Whereas many of upregulated genes could be the result of direct or indirect regulation by *SPL*-dependent pathways, the fact that in some cases the misexpression of some of them is conserved in the unrelated meiotic mutant *dif1* suggests that in wild-type ovules their repression is dependent on the presence of a developing or differentiated female gametophyte. Transcription factors misexpressed in both *spl* and *dif1* include a homeodomain gene of the *KNOX/BELL* family: *BLH1*. In *Arabidopsis*, misexpression of the *BEL1*-like homeodomain 1 gene (*BLH1*) in the female gametophyte results in the *eostre* phenotype, a cell fate switch from synergid to egg cell [42], suggesting that suppression of *TALE* genes is essential for normal development and cell specification in the female gametophyte. BEL1-like proteins are known to interact with distinct TALE members of the KNOX homeodomain family through the KNOX-MEINOX domain, binding to DNA as a heterodimer [52], [53]. The *eostre* phenotype depends on the activity of the class II KNOX gene KNAT3, and is partially phenocopied by loss-of-function of the TALE-interacting OVATE protein AtOFP5 [42], suggesting that that repression of the BLH1-KNAT3 dimer activity by AtOFP5 is essential for cell specification in the female gametophyte. Similar TALE homeodomain genes promote the establishment of a zygotic program in vegetative cells of *Chlamydomonas reinhardtii*, regulating its haploid-diploid transition and providing evidence of a shared common ancestry with proteins regulating meristem specification in land plants [54]. Interestingly, GSM1 is the *C. reinhardtii* homolog of KNAT3 [54]. Our results extend this perspective by suggesting that in some cases the female gametophyte is responsible for suppressing the expression of TALE proteins, a complex mechanism that we speculate could contribute to impede the establishment of a sporophytic phase in the ovule before double fertilization.

Whereas determining if the ectopic sporophytic activity of *BLH1* is detrimental to female gametogenesis will require additional experimental work, our results show that the misexpression of *IOR* in sporophytic tissues of the ovule impedes the progression of female gametogenesis and maintains the presence of the normally degenerating L1 layer. *IOR* encodes a WD40 transducin repeat protein likely involved in meristem development [55], but for which a precise function remains to be elucidated. Several WD40 genes that include *WD repeat 55 (WD55), LACHESIS (LIS), SLOW WALKER1 (SWA1), MULTICOPY SUPRESSOR OF IRA1* (*MSI1*), *ANAPHASE-PROMOTING COMPLEX4 (APC4)*, *YAO*, and *NOF1* are known to be involved in female gametophyte development and sexual reproduction. Mutations in *WD55* cause failure in the fusion of the polar nuclei and arrest of both embryo and endosperm development [56], *SWA1* is expressed in cells undergoing active division and encodes a protein with six WD40 repeats that is localized in the nucleolus; mutations in *SWA1* result in female gametophyte arrest at variable stages [57].*YAO* encodes a nucleolar WD40-repeat component of the U3 small nucleolar RNP complex that is critical for gametogenesis and embryogenesis [58]. *APC4* encodes a E3 ubiquitin ligase that is also involved in regulating gametophytic cell-cycle progression [59]. *NOF1* is involved in the control of rRNA expression, and *nof1* mutants show arrest in female gametogenesis [60]. *LIS,* that encodes a homolog of the yeast PRP4 protein that is associated with the U4/U6 spliceosome complex, restricts egg cell fate in the female gametophyte [61]. Finally, *MSI1* encodes a member of the evolutionarily conserved *Polycomb* group (PcG) chromatin-remodeling complex that is homologous to the Retinoblastoma binding proteins P55 in *Drosophila* and RbAp48 in mammals. Mutations in *MSI1* induce the formation of parthenogenetic embryos [62].

Whereas both *CaMV35S*- and *pNUC1*-driven misexpression of *IOR* results in arrest of female gametogenesis after normal meiosis and differentiation of the functional megaspore, misexpression of *IOR* in the female gametophyte does not affect fertility or gametogenesis. This combination of results indicates that the gametophytic detrimental effect caused by *IOR* expression is dependent on either *IOR* molecular interactions with factors present in the sporophyte but not the female gametophyte, or on *IOR* posttranscriptional modifications that are dependent on *IOR* expression in the sporophytic tissues of the ovule. In the absence of a female gametophyte, *IOR* mRNA is preferentially localized in the L1 layer but is also present in the rest of the ovule, suggesting that the detrimental effect over female gametophyte formation might involve a cross-talk communication over several sporophytic cell layers. Our overall results indicate that the female gametophyte can repress the activity of specific genes detrimental for its growth and development, offering a large-scale quantitative reference of global expression in ovules lacking a female gametophyte that can be used for genomic and developmental studies of the sporophytic-to-gametophytic transition in flowering plants.

## Supporting Information

Figure S1 **RT-PCR analysis in wild type and** ***spl*** **ovules.**
(PDF)

Figure S2 **GUS expression in vegetative and reproductive organs of** ***pAt4g16190::GUS*** **and** ***pAt2g46680::GUS*** **transformants.**
(PDF)

Figure S3 **Isolation and activity of the pNUC1 and pES1 promoters.**
(PDF)

Figure S4 **Female gametophyte development in wild-type ovules of Arabidopsis.**
(PDF)

Table S1 **List of MPSS differentially expressed genes upregulated in** ***spl*** **ovules.**
(PDF)

Table S2 **Pfam analysis of 1335 upregulated genes in** ***spl*** **ovules.**
(PDF)

Table S3 **Phenotypic quantification in At1g47610 and At2g46680 transformed lines using the** **CaMV35S, pNUC or pES1 promoter.**
(PDF)

Table S4 **Quantification of reduced fertility in At1g47610 and At2g46680 selected transformant lines.**
(PDF)

Table S5 **Gene expression comparison between MPSS and ATH1 microarray results.**
(PDF)

Table S6 **Primers used to analyze expression of candidate genes in** ***spl*** **and wild-type ovules.**
(PDF)
