# Supplementary material for: Functional Analysis of Sporophytic Transcripts Repressed by the Female Gametophyte in the Ovule of Arabidopsis thaliana
Source: PLoS One. 2013 Oct 23;8(10):e76977. doi: 10.1371/journal.pone.0076977 (PMC3806734; doi:10.1371/journal.pone.0076977)
Supplement: Figure S1 — RT-PCR analysis in wild type and spl ovules. (PDF) [file pone.0076977.s001.pdf]

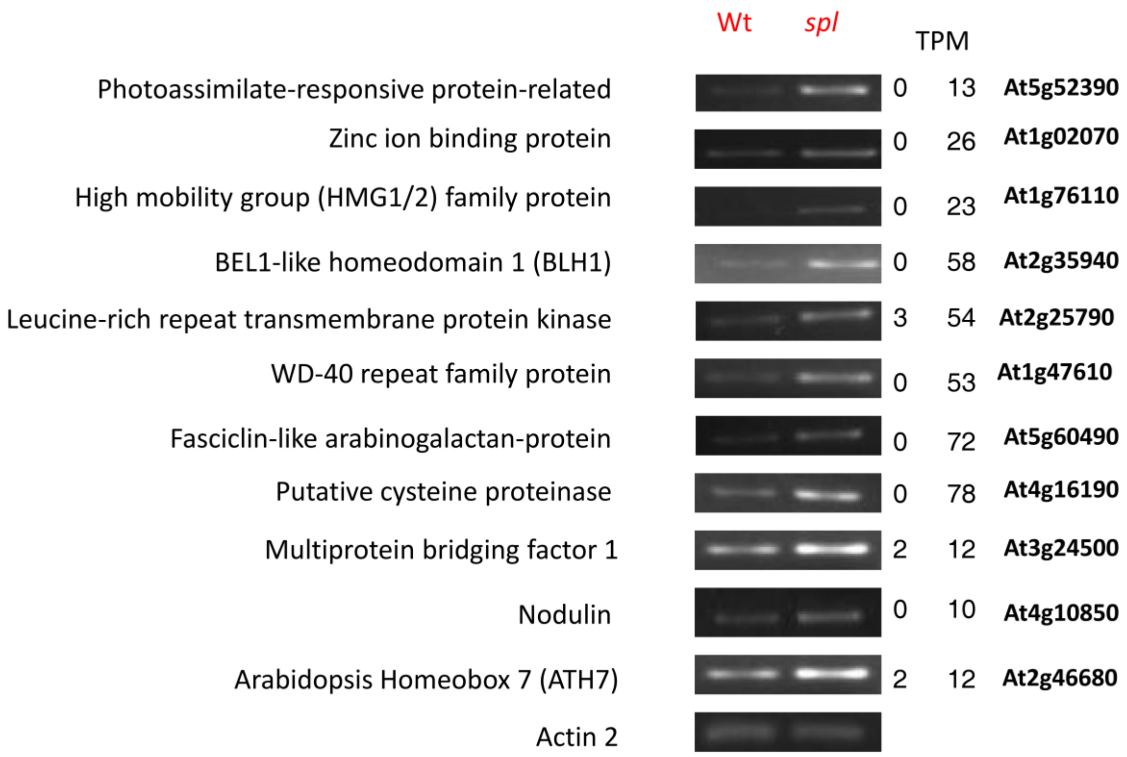

Figure S1. RT-PCR analysis in wild type and *spl* ovules.
