# Supplementary material for: Functional Analysis of Sporophytic Transcripts Repressed by the Female Gametophyte in the Ovule of Arabidopsis thaliana
Source: PLoS One. 2013 Oct 23;8(10):e76977. doi: 10.1371/journal.pone.0076977 (PMC3806734; doi:10.1371/journal.pone.0076977)
Supplement: Figure S2 — GUS expression in vegetative and reproductive organs of pAt4g16190::GUS and pAt2g46680::GUS transformants. (PDF) [file pone.0076977.s002.pdf]

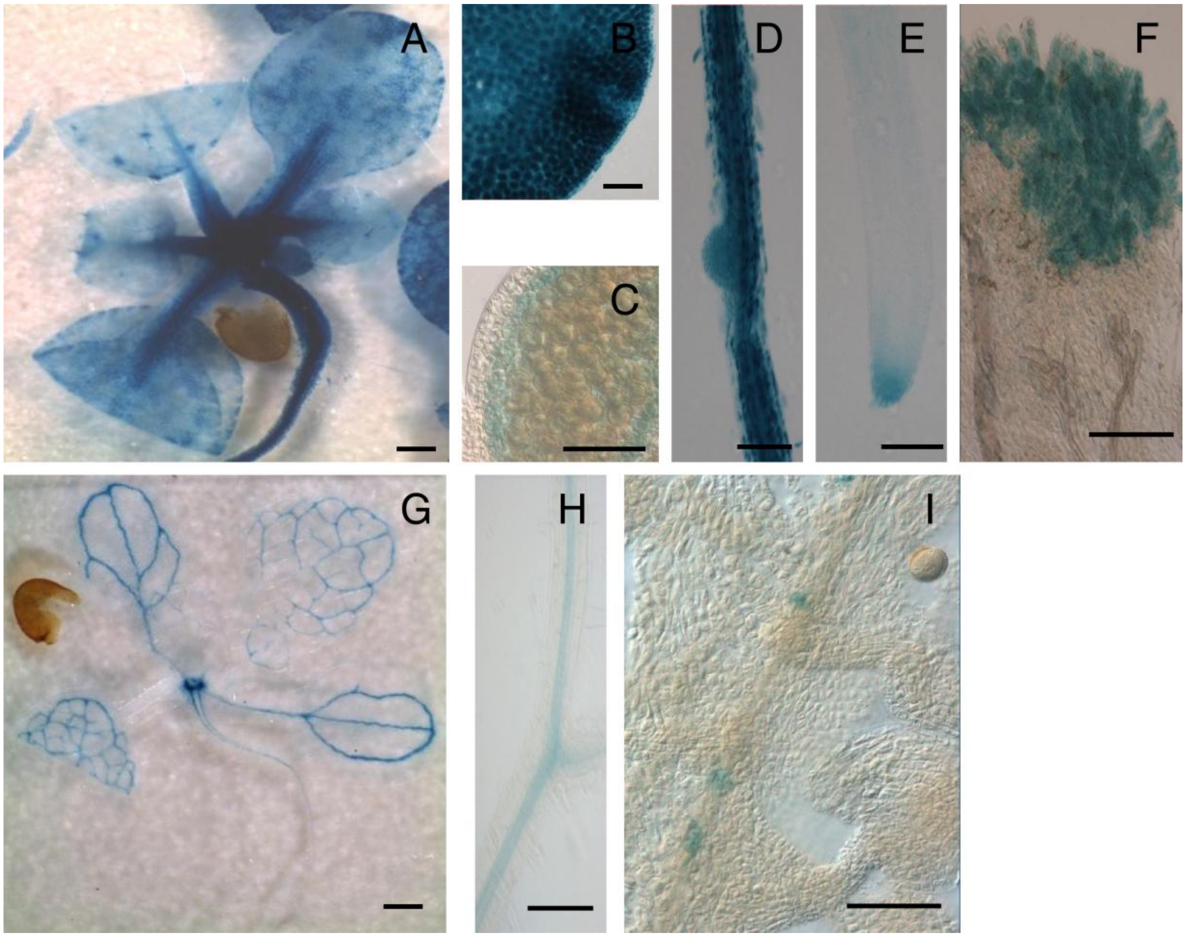

Figure S2. GUS expression in vegetative and reproductive organs of *pAt4g16190::GUS* and *pAt2g46680::GUS transformants*.

(A) *pAt4g16190::GUS* expression in wild type seedlings nine days after germination.

(B) *pAt4g16190::GUS* expression in cotyledons of seedlings seven days after germination.

(C) *pAt4g16190::GUS* expression in anthers at mature pollen stage.

(D) *pAt4g16190::GUS* expression in roots nine days after germination.

(E) *pAt4g16190::GUS* expression in roots tips nine days after germination.

(F) *pAt4g16190::GUS* expression in stigma of wild type gynoecia.

(G) *pAt2g46680::GUS* expression in seedlings nine days after germination.

(H) *pAt2g46680::GUS* expression in roots nine days after germination.

Sale bars: 50 μm.

(I) *pAt2g46680::GUS* expression in the placenta.

Scale bars: 50 μm.
