# Supplementary material for: Functional Analysis of Sporophytic Transcripts Repressed by the Female Gametophyte in the Ovule of Arabidopsis thaliana
Source: PLoS One. 2013 Oct 23;8(10):e76977. doi: 10.1371/journal.pone.0076977 (PMC3806734; doi:10.1371/journal.pone.0076977)
Supplement: Figure S3 — Isolation and activity of the pNUC1 and pES1 promoters. (PDF) [file pone.0076977.s003.pdf]

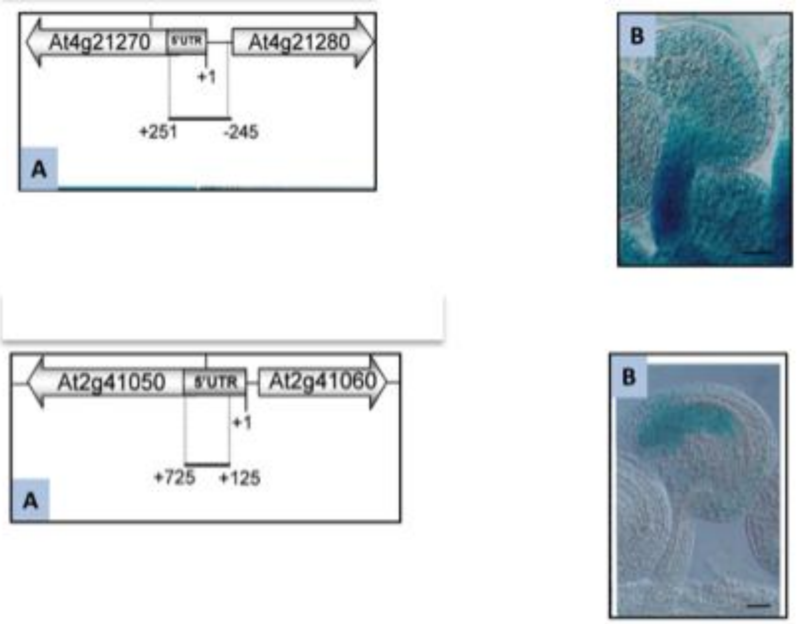

Figure S3. Isolation and activity of the *pNUC*1 and *pES1* promoters.

<u>Isolation of pNUC.</u>

(**A**) A 495 bp fragment was amplified from nucleotide -495 to -1 upstream of the At4g21270 gene.

(**B**) GUS expression in the ovule of *pNUC1::GUS* transformants.

Scale bar: 20 µm.

<u>Isolation of pES1.</u>

(**A**) A 600 bp fragment was amplified from nucleotide –597 to –3 upstream of the transcriptional initiation site of the At2g41050 gene.

(**B**) GUS expression in the ovule of *pES1::GUS* transformants

Scale bar: 20 µm.
