# Supplementary material for: Functional Analysis of Sporophytic Transcripts Repressed by the Female Gametophyte in the Ovule of Arabidopsis thaliana
Source: PLoS One. 2013 Oct 23;8(10):e76977. doi: 10.1371/journal.pone.0076977 (PMC3806734; doi:10.1371/journal.pone.0076977)
Supplement: Figure S4 — Female gametophyte development in wild-type ovules of Arabidopsis. (PDF) [file pone.0076977.s004.pdf]

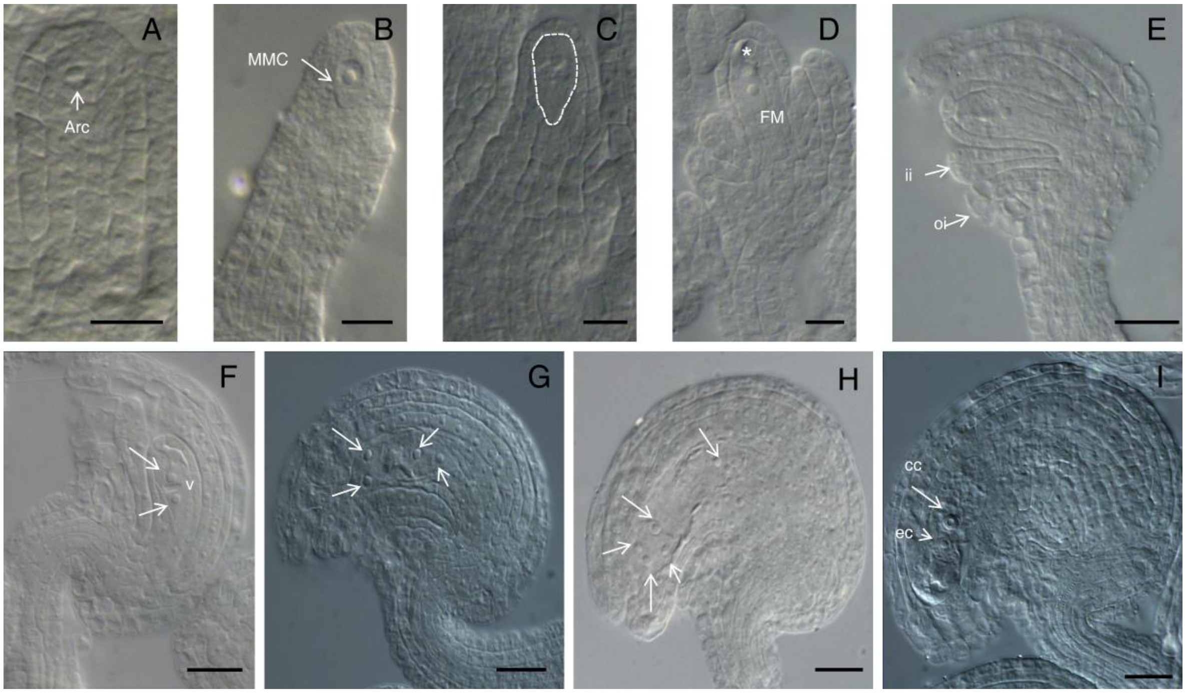

Figure S4. Female gametophyte development in wild-type ovules of Arabidopsis.

(**A**) Differentiation of archeosporial (Arc) cell in a pre-meiotic ovule.

(**B**) and (**C**) Pre-meiotic ovule showing a megaspore mother cell (MMC).

(D) Pre-meiotic ovule showing a MMC (dashed) initating meiosis.

(**E**) Differentiation of inner (ii) and outer (oi) integuments in a wild-type ovule.

(**F**) Post-meiotic ovule at two-nuclear stage of female gametogenesis; nuclei are highlighted with arrows (v=vacuole).

(**G**) Ovule at the four-nuclear stage of female gametogenesis (arrows).

(**H**) Ovule in the eight-nuclear stage of gametogenesis (arrows).

(**I**) Ovule containing a cellularized female gametophyte; cc= central cell: ec= egg cell (ec).

Scale bars, A-D: 10 μm; E-I, 20 μm.
