# Supplementary material for: Functional Analysis of Sporophytic Transcripts Repressed by the Female Gametophyte in the Ovule of Arabidopsis thaliana
Source: PLoS One. 2013 Oct 23;8(10):e76977. doi: 10.1371/journal.pone.0076977 (PMC3806734; doi:10.1371/journal.pone.0076977)
Supplement: Table S1 — List of MPSS differentially expressed genes upregulated in spl ovules. (PDF) [file pone.0076977.s005.pdf]

Table S1. **List of MPSS differentially expressed genes upregulated in *spl* ovules**. The table includes the values of the Fischer exact test for all 1517 genes upregulated in spl ovules.

Gene	wt	*spl*	Fisher p value
AT1G55040	2	15	2.35E-03
AT1G64690	2	15	2.35E-03
AT2G37930	2	15	2.35E-03
AT3G49710	2	15	2.35E-03
AT5G20490	6	23	2.32E-03
AT3G10640	17	41	2.23E-03
AT5G49440	17	41	2.23E-03
AT5G48220	21	47	2.19E-03
AT4G35987	7	25	2.10E-03
AT2G41720	8	27	1.88E-03
AT1G08600	1	13	1.83E-03
AT1G22800	1	13	1.83E-03
AT2G18440	1	13	1.83E-03
AT3G26730	1	13	1.83E-03
AT3G26780	1	13	1.83E-03
AT4G17310	1	13	1.83E-03
AT4G38970	1	13	1.83E-03
AT5G63320	1	13	1.83E-03
AT5G10510	12	34	1.64E-03
AT4G32970	17	42	1.55E-03
AT2G29690	4	20	1.54E-03
AT1G25320	5	22	1.51E-03
AT3G53490	5	22	1.51E-03
AT5G13720	5	22	1.51E-03
AT1G63010	3	18	1.49E-03
AT2G02790	3	18	1.49E-03
AT4G16807	3	18	1.49E-03
AT5G63780	3	18	1.49E-03
AT1G25500	2	16	1.31E-03
AT1G28960	2	16	1.31E-03
AT2G26180	2	16	1.31E-03
AT4G03070	2	16	1.31E-03
AT4G15040	2	16	1.31E-03
AT4G23840	2	16	1.31E-03
AT1G01780	1	14	9.77E-04
AT1G61960	1	14	9.77E-04
AT1G68460	1	14	9.77E-04
AT2G38420	1	14	9.77E-04
AT5G05640	1	14	9.77E-04
AT5G35370	1	14	9.77E-04
AT5G44200	1	14	9.77E-04
AT5G63290	1	14	9.77E-04

AT4G29440	18	45	8.98E-04
AT4G35540	6	25	8.78E-04
ATMG00090	6	25	8.78E-04
AT1G33265	3	19	8.56E-04
AT3G17340	3	19	8.56E-04
AT5G63280	3	19	8.56E-04
AT4G01990	11	34	8.24E-04
AT4G35130	7	27	8.22E-04
AT2G47390	14	39	8.03E-04
AT1G22250	8	29	7.53E-04
AT4G15730	17	44	7.30E-04
AT1G52640	2	17	7.29E-04
AT2G04530	2	17	7.29E-04
AT2G33847	2	17	7.29E-04
AT2G40435	2	17	7.29E-04
AT4G08580	2	17	7.29E-04
AT5G20360	2	17	7.29E-04
AT5G53830	2	17	7.29E-04
AT1G63240	12	36	7.17E-04
AT1G05350	9	31	6.80E-04
AT3G58530	18	46	6.18E-04
AT1G68780	5	24	5.46E-04
AT3G21500	5	24	5.46E-04
AT3G51480	5	24	5.46E-04
AT4G04180	5	24	5.46E-04
AT3G60370	11	35	5.36E-04
AT1G58350	6	26	5.35E-04
AT2G30900	1	15	5.19E-04
AT5G03900	1	15	5.19E-04
AT5G56100	1	15	5.19E-04
AT1G53280	7	28	5.08E-04
AT1G64440	7	28	5.08E-04
AT3G18620	3	20	4.88E-04
AT4G14490	3	20	4.88E-04
AT4G38920	3	20	4.88E-04
AT2G13650	8	30	4.72E-04
AT3G28340	12	37	4.70E-04
AT4G00230	15	42	4.60E-04
AT1G69280	9	32	4.31E-04
AT3G52890	9	32	4.31E-04
AT3G46590	10	34	3.88E-04
AT5G08620	11	36	3.46E-04
AT2G46060	5	25	3.25E-04
AT3G61415	5	25	3.25E-04
AT5G12940	7	29	3.13E-04
AT1G34750	4	23	3.11E-04
AT2G30040	4	23	3.11E-04
AT3G53930	4	23	3.11E-04

AT5G41110	4	23	3.11E-04
AT5G53900	8	31	2.94E-04
AT1G09520	18	48	2.87E-04
AT1G10657	3	21	2.77E-04
AT1G54040	3	21	2.77E-04
AT2G13600	3	21	2.77E-04
AT2G27810	3	21	2.77E-04
AT2G45910	3	21	2.77E-04
AT5G40942	3	21	2.77E-04
AT1G53190	1	16	2.75E-04
AT2G26250	1	16	2.75E-04
AT4G05040	1	16	2.75E-04
AT4G07990	1	16	2.75E-04
AT4G12640	1	16	2.75E-04
AT4G17380	1	16	2.75E-04
AT5G24000	1	16	2.75E-04
AT5G64330	1	16	2.75E-04
AT1G77740	23	56	2.64E-04
AT5G45030	21	53	2.57E-04
AT1G80550	2	19	2.21E-04
AT2G12550	2	19	2.21E-04
AT2G41250	2	19	2.21E-04
AT5G06000	2	19	2.21E-04
AT5G66005	2	19	2.21E-04
AT3G21330	6	28	1.95E-04
AT3G22435	7	30	1.91E-04
AT3G48100	4	24	1.80E-04
AT5G41920	4	24	1.80E-04
AT1G19950	13	41	1.75E-04
AT5G04040	13	41	1.75E-04
AT1G74160	9	34	1.70E-04
AT1G49910	3	22	1.57E-04
AT5G53450	3	22	1.57E-04
AT5G57610	22	56	1.50E-04
AT2G36630	1	17	1.45E-04
AT3G10570	1	17	1.45E-04
AT3G14330	1	17	1.45E-04
AT1G09940	11	38	1.42E-04
AT2G32090	15	45	1.35E-04
AT2G03690	12	40	1.28E-04
AT2G46680	2	20	1.21E-04
AT3G51030	21	55	1.21E-04
AT2G39660	6	29	1.17E-04
AT1G21380	13	42	1.14E-04
AT2G22430	8	33	1.12E-04
AT4G25170	8	33	1.12E-04
AT1G15300	9	35	1.06E-04
AT5G03930	9	35	1.06E-04

AT1G53530	4	25	1.04E-04
AT1G56710	4	25	1.04E-04
AT5G19221	4	25	1.04E-04
AT5G20990	4	25	1.04E-04
AT3G03360	17	49	1.02E-04
AT3G02350	11	39	9.03E-05
AT1G45207	3	23	8.80E-05
AT4G16265	3	23	8.80E-05
AT4G30850	3	23	8.80E-05
AT1G08460	1	18	7.63E-05
AT1G21590	1	18	7.63E-05
AT5G45620	1	18	7.63E-05
AT3G27210	7	32	7.03E-05
AT2G32295	6	30	6.96E-05
AT3G05190	6	30	6.96E-05
AT2G17990	8	34	6.88E-05
AT4G11160	5	28	6.62E-05
AT1G19110	2	21	6.61E-05
AT1G19140	2	21	6.61E-05
AT4G31860	2	21	6.61E-05
AT5G11760	2	21	6.61E-05
AT5G51110	2	21	6.61E-05
AT2G38695	11	40	5.71E-05
AT2G24860	12	42	5.21E-05
AT3G60680	12	42	5.21E-05
AT1G22070	3	24	4.92E-05
AT5G15170	20	56	4.37E-05
AT2G15530	6	31	4.13E-05
ATMG01370	6	31	4.13E-05
AT2G37860	1	19	4.01E-05
AT5G06280	1	19	4.01E-05
AT5G17530	1	19	4.01E-05
AT5G51545	5	29	3.86E-05
AT5G54940	5	29	3.86E-05
AT1G05620	15	48	3.76E-05
AT5G35970	26	66	3.67E-05
AT2G25290	2	22	3.59E-05
AT3G08710	2	22	3.59E-05
AT3G03750	11	41	3.59E-05
AT3G49645	11	41	3.59E-05
AT3G13380	4	27	3.40E-05
AT4G24810	4	27	3.40E-05
AT3G08510	12	43	3.31E-05
AT3G60130	12	43	3.31E-05
AT1G51745	27	68	3.12E-05
AT5G51440	27	68	3.12E-05
AT3G06980	13	45	3.01E-05
AT3G25780	25	65	2.97E-05

AT4G15520	17	52	2.93E-05
AT1G05300	3	25	2.74E-05
AT1G55300	3	25	2.74E-05
AT3G15605	3	25	2.74E-05
AT1G77850	9	38	2.49E-05
AT2G35360	15	49	2.44E-05
ATCG00810	10	40	2.39E-05
AT3G27890	43	93	2.17E-05
AT2G35710	1	20	2.10E-05
AT2G47310	1	20	2.10E-05
AT3G04240	1	20	2.10E-05
AT4G22340	1	20	2.10E-05
AT5G04590	1	20	2.10E-05
AT3G12380	12	44	2.09E-05
AT2G01620	2	23	1.94E-05
AT2G33310	2	23	1.94E-05
AT3G03780	2	23	1.94E-05
AT2G35350	4	28	1.93E-05
AT5G64600	4	28	1.93E-05
AT5G48240	13	46	1.92E-05
AT3G06200	8	37	1.54E-05
AT5G54920	8	37	1.54E-05
AT1G56140	3	26	1.52E-05
AT3G60660	3	26	1.52E-05
AT4G19110	9	39	1.52E-05
AT4G23180	9	39	1.52E-05
AT5G20320	9	39	1.52E-05
AT5G08360	7	35	1.51E-05
AT1G67170	10	41	1.47E-05
AT1G71720	10	41	1.47E-05
AT1G54180	6	33	1.43E-05
AT3G58620	6	33	1.43E-05
AT1G42960	11	43	1.40E-05
AT3G12950	12	45	1.31E-05
AT1G51460	20	59	1.30E-05
AT1G02320	13	47	1.22E-05
AT1G23860	13	47	1.22E-05
AT2G43990	13	47	1.22E-05
AT1G65450	14	49	1.11E-05
AT1G62981	1	21	1.10E-05
AT3G26920	1	21	1.10E-05
AT5G03350	1	21	1.10E-05
AT5G25490	1	21	1.10E-05
AT5G47490	1	21	1.10E-05
AT5G07630	4	29	1.09E-05
AT1G30680	9	40	9.27E-06
AT3G27870	9	40	9.27E-06
AT1G26330	16	53	9.10E-06

AT2G43020	7	36	8.97E-06
AT3G20670	7	36	8.97E-06
AT2G23890	11	44	8.70E-06
AT3G51000	11	44	8.70E-06
AT1G01670	3	27	8.43E-06
AT3G27283	3	27	8.43E-06
AT5G58787	3	27	8.43E-06
AT5G19420	5	32	7.43E-06
AT5G45780	5	32	7.43E-06
AT5G49230	5	32	7.43E-06
AT1G52630	1	22	5.72E-06
AT5G18790	20	61	5.66E-06
AT2G15320	2	25	5.65E-06
AT3G12670	9	41	5.62E-06
AT1G03720	7	37	5.30E-06
AT2G34570	12	47	5.13E-06
AT4G11560	46	102	4.81E-06
AT5G52230	3	28	4.65E-06
AT4G27940	5	33	4.26E-06
AT5G51980	5	33	4.26E-06
AT3G25230	15	53	4.12E-06
AT1G02110	16	55	3.76E-06
AT4G34920	4	31	3.47E-06
AT5G50012	4	31	3.47E-06
AT1G01240	8	40	3.31E-06
AT2G18900	8	40	3.31E-06
AT4G37300	11	46	3.31E-06
AT1G31930	27	74	3.18E-06
AT3G59050	2	26	3.03E-06
AT5G28010	2	26	3.03E-06
AT2G32440	13	50	3.02E-06
AT2G36310	1	23	2.98E-06
AT3G18370	1	23	2.98E-06
AT5G12030	1	23	2.98E-06
AT5G15920	1	23	2.98E-06
AT1G17920	22	66	2.88E-06
AT1G49820	22	66	2.88E-06
AT2G33820	3	29	2.56E-06
AT3G26370	31	81	2.53E-06
AT2G34670	11	47	2.03E-06
AT3G05100	11	47	2.03E-06
AT4G10630	30	80	2.02E-06
AT1G16680	8	41	1.97E-06
AT2G34730	8	41	1.97E-06
AT3G06520	18	60	1.97E-06
AT4G19440	7	39	1.83E-06
AT2G42400	14	53	1.77E-06
AT1G75310	2	27	1.62E-06

AT3G56820	2	27	1.62E-06
AT2G27080	20	64	1.59E-06
AT5G52010	20	64	1.59E-06
AT1G12200	1	24	1.55E-06
AT3G27416	3	30	1.40E-06
AT3G57000	3	30	1.40E-06
AT4G01730	17	59	1.40E-06
AT4G27470	11	48	1.24E-06
AT3G26030	9	44	1.22E-06
AT5G49530	12	50	1.22E-06
AT1G52140	13	52	1.17E-06
AT4G09900	8	42	1.16E-06
AT3G19210	14	54	1.11E-06
AT4G28770	15	56	1.04E-06
AT5G03690	17	60	8.92E-07
AT1G08960	2	28	8.68E-07
AT1G53790	2	28	8.68E-07
AT1G60080	2	28	8.68E-07
AT2G16480	1	25	8.05E-07
AT3G05675	1	25	8.05E-07
AT4G16450	1	25	8.05E-07
AT5G48610	1	25	8.05E-07
AT1G11410	3	31	7.66E-07
AT5G27200	3	31	7.66E-07
AT3G09100	10	47	7.52E-07
AT3G04160	26	76	7.45E-07
AT1G14970	9	45	7.29E-07
AT2G35040	13	53	7.24E-07
AT2G37970	8	43	6.87E-07
AT1G67560	6	39	5.42E-07
AT5G16870	6	39	5.42E-07
AT1G32870	2	29	4.63E-07
AT1G53560	2	29	4.63E-07
AT3G58460	2	29	4.63E-07
AT2G45030	12	52	4.57E-07
AT4G35440	12	52	4.57E-07
AT3G16980	28	2	4.51E-07
AT2G17265	13	54	4.47E-07
AT3G22270	5	37	4.44E-07
AT5G10050	5	37	4.44E-07
AT2G17710	35	92	4.37E-07
AT1G78010	9	46	4.34E-07
AT5G46210	9	46	4.34E-07
AT4G15450	1	26	4.17E-07
AT4G21990	8	44	4.04E-07
AT2G45740	4	35	3.35E-07
AT4G15420	18	64	3.32E-07
AT1G19835	37	96	3.20E-07

AT1G77550	6	40	3.10E-07
AT5G51370	6	40	3.10E-07
AT5G10940	42	104	3.00E-07
AT1G15060	11	51	2.78E-07
AT2G21280	11	51	2.78E-07
AT4G23640	20	68	2.77E-07
AT4G13590	10	49	2.71E-07
AT5G53010	10	49	2.71E-07
AT5G60370	5	38	2.50E-07
AT1G03260	8	45	2.37E-07
AT1G08640	34	92	2.37E-07
AT3G05940	3	33	2.27E-07
AT4G37870	3	33	2.27E-07
AT1G69070	17	63	2.27E-07
AT1G61030	1	27	2.16E-07
AT1G80170	1	27	2.16E-07
AT3G19570	1	27	2.16E-07
AT3G20790	18	65	2.11E-07
AT3G24560	7	43	2.10E-07
AT4G17090	7	43	2.10E-07
AT1G09700	4	36	1.86E-07
AT1G73680	4	36	1.86E-07
AT5G46760	4	36	1.86E-07
AT5G10380	6	41	1.77E-07
AT3G14010	12	54	1.70E-07
AT5G15820	12	54	1.70E-07
AT3G07230	11	52	1.67E-07
AT2G46400	10	50	1.62E-07
AT5G46570	10	50	1.62E-07
AT1G07640	5	39	1.41E-07
AT4G31800	8	46	1.39E-07
AT1G50640	18	66	1.33E-07
AT1G06510	2	31	1.31E-07
AT1G75410	2	31	1.31E-07
AT2G26150	27	82	1.26E-07
AT4G10140	19	68	1.24E-07
AT3G26980	20	70	1.14E-07
AT5G23710	39	102	1.11E-07
AT4G38120	6	42	1.01E-07
AT2G29890	11	53	1.01E-07
AT1G76970	16	63	9.45E-08
AT3G07020	8	47	8.07E-08
AT2G37070	5	40	7.88E-08
AT1G04760	7	45	6.98E-08
AT1G01660	2	32	6.94E-08
AT2G43690	3	35	6.68E-08
AT5G44780	3	35	6.68E-08
AT4G19860	15	62	6.09E-08

AT2G35790	11	54	6.03E-08
AT3G59800	11	54	6.03E-08
AT1G23090	6	43	5.73E-08
AT5G55896	23	77	5.52E-08
AT1G53390	9	50	5.27E-08
AT2G45440	9	50	5.27E-08
AT1G73060	39	104	5.19E-08
AT1G29040	24	79	4.99E-08
AT3G61350	7	46	4.01E-08
AT5G14260	30	90	3.78E-08
AT1G73740	2	33	3.67E-08
AT3G52770	2	33	3.67E-08
AT3G62040	2	33	3.67E-08
AT1G27752	10	53	3.39E-08
AT2G16850	4	39	3.11E-08
AT5G60120	4	39	3.11E-08
AT5G17010	9	51	3.09E-08
AT5G58150	9	51	3.09E-08
AT1G26660	1	30	2.98E-08
AT4G31060	1	30	2.98E-08
AT1G54120	14	62	2.33E-08
AT3G51890	7	47	2.29E-08
AT3G21295	11	56	2.15E-08
AT1G72320	24	81	2.08E-08
AT2G06210	19	72	1.97E-08
AT2G31170	19	72	1.97E-08
AT2G28105	2	34	1.94E-08
AT2G34920	2	34	1.94E-08
AT5G60050	6	45	1.83E-08
AT3G48430	8	50	1.57E-08
AT4G32620	8	50	1.57E-08
AT1G14000	27	87	1.53E-08
AT3G18770	16	67	1.39E-08
AT4G38070	43	114	1.32E-08
AT1G31350	7	48	1.31E-08
AT1G76460	20	75	1.17E-08
AT1G67230	3	38	1.05E-08
AT1G77990	4	41	9.34E-09
AT3G54050	4	41	9.34E-09
AT1G15720	15	66	8.64E-09
AT3G01300	18	72	8.08E-09
AT3G60400	5	44	7.60E-09
AT1G67700	9	54	6.11E-09
AT3G17120	9	54	6.11E-09
AT1G07380	6	47	5.81E-09
AT1G78620	6	47	5.81E-09
AT1G14680	3	39	5.64E-09
AT5G08200	3	39	5.64E-09

AT4G09010	2	36	5.40E-09
AT4G25730	15	67	5.26E-09
AT3G12830	16	69	5.25E-09
AT3G11210	17	71	5.16E-09
AT1G34160	4	42	5.10E-09
AT1G01630	13	63	5.05E-09
AT1G04200	12	61	4.81E-09
AT4G19660	7	50	4.24E-09
AT1G19397	5	45	4.21E-09
AT1G56345	10	57	4.05E-09
AT3G11320	28	92	3.78E-09
AT5G48120	9	55	3.55E-09
AT4G11280	6	48	3.26E-09
AT3G15400	3	40	3.02E-09
AT5G48880	3	40	3.02E-09
AT3G26085	8	53	2.99E-09
AT2G26170	2	37	2.84E-09
AT4G21210	2	37	2.84E-09
AT5G03210	2	37	2.84E-09
AT2G30280	7	51	2.40E-09
AT4G19950	7	51	2.40E-09
AT1G17410	5	46	2.33E-09
AT2G22990	5	46	2.33E-09
AT2G44310	5	46	2.33E-09
AT4G12620	5	46	2.33E-09
AT1G79950	1	34	2.10E-09
AT3G14415	1	34	2.10E-09
AT4G36648	30	97	1.99E-09
AT1G64980	46	124	1.84E-09
AT1G12780	6	49	1.82E-09
AT5G48450	13	65	1.81E-09
AT2G44760	8	54	1.71E-09
AT1G76050	12	63	1.70E-09
AT3G12250	3	41	1.62E-09
AT5G38890	3	41	1.62E-09
AT3G57510	4	44	1.52E-09
AT3G11490	23	85	1.52E-09
AT1G20670	2	38	1.49E-09
AT3G13530	10	59	1.38E-09
AT1G12730	1	35	1.08E-09
AT3G06530	1	35	1.08E-09
AT1G48830	8	55	9.77E-10
AT3G16260	8	55	9.77E-10
AT5G57930	11	62	9.10E-10
AT5G05350	37	111	8.77E-10
AT4G02510	3	42	8.66E-10
AT5G17900	34	106	8.32E-10
AT1G15860	4	45	8.23E-10

AT1G23050	2	39	7.85E-10
AT2G38460	2	39	7.85E-10
AT3G61320	2	39	7.85E-10
AT4G16380	38	113	7.73E-10
AT1G15250	7	53	7.68E-10
AT1G21065	91	195	7.42E-10
AT4G18300	14	69	6.81E-10
AT2G34650	62	152	6.64E-10
AT1G61210	13	67	6.43E-10
AT2G25800	6	51	5.68E-10
AT1G26110	1	36	5.53E-10
AT1G56660	1	36	5.53E-10
AT4G21060	1	36	5.53E-10
AT4G23070	1	36	5.53E-10
AT3G03180	11	63	5.33E-10
AT5G56600	10	61	4.65E-10
AT3G01510	3	43	4.63E-10
AT4G27657	15	72	4.27E-10
AT1G21570	22	86	4.00E-10
AT2G37560	13	68	3.81E-10
AT3G15070	23	88	3.80E-10
AT5G36250	24	90	3.59E-10
AT2G39670	6	52	3.16E-10
AT1G17870	10	62	2.69E-10
AT1G76440	14	71	2.43E-10
AT4G00930	4	47	2.42E-10
AT4G29140	23	89	2.39E-10
AT3G28200	34	109	2.34E-10
AT3G15220	13	69	2.26E-10
AT5G66950	13	69	2.26E-10
AT2G27340	2	41	2.15E-10
AT4G08290	2	41	2.15E-10
AT4G17550	5	50	2.14E-10
AT5G61760	5	50	2.14E-10
AT5G06460	61	154	1.84E-10
AT3G48760	8	58	1.80E-10
AT3G56400	6	53	1.76E-10
AT4G37270	6	53	1.76E-10
AT2G44090	57	148	1.65E-10
AT3G05130	29	101	1.59E-10
AT4G18840	1	38	1.46E-10
AT5G17440	1	38	1.46E-10
AT5G49660	1	38	1.46E-10
AT3G16950	13	70	1.34E-10
AT3G44260	13	70	1.34E-10
AT3G13690	39	119	1.32E-10
AT1G21640	3	45	1.31E-10
AT4G29780	4	48	1.31E-10

AT3G63290	9	61	1.29E-10
AT5G04110	26	96	1.26E-10
AT1G24610	2	42	1.13E-10
AT1G42430	2	42	1.13E-10
AT2G33800	2	42	1.13E-10
AT4G12990	2	42	1.13E-10
AT1G53850	20	85	1.01E-10
AT5G24850	20	85	1.01E-10
AT1G77120	21	87	9.93E-11
AT4G22580	21	87	9.93E-11
AT1G70190	10	64	8.97E-11
AT2G25050	13	71	7.88E-11
AT4G33760	7	57	7.65E-11
AT3G11964	37	117	7.19E-11
AT4G14890	3	46	6.99E-11
AT4G29170	65	163	6.82E-11
AT1G31835	5	52	6.41E-11
AT2G34630	20	86	6.21E-11
AT1G71880	42	126	6.03E-11
AT3G16910	28	102	4.44E-11
AT5G43745	7	58	4.28E-11
AT1G49520	3	47	3.71E-11
AT2G34770	5	53	3.50E-11
AT5G49980	24	95	3.48E-11
AT3G21420	16	79	3.47E-11
AT3G12270	25	97	3.33E-11
AT1G03520	15	77	3.27E-11
AT3G51110	14	75	3.02E-11
AT1G43620	6	56	2.98E-11
AT3G13490	7	59	2.39E-11
AT4G39850	18	84	2.26E-11
AT1G22710	23	94	2.24E-11
AT2G32900	37	120	2.00E-11
AT1G50730	3	48	1.97E-11
AT1G62422	3	48	1.97E-11
AT5G56010	5	54	1.91E-11
AT1G65560	45	134	1.83E-11
AT5G62570	28	104	1.78E-11
AT5G10560	10	67	1.70E-11
AT5G14960	2	45	1.61E-11
AT2G46810	31	110	1.44E-11
AT5G24800	17	83	1.31E-11
AT3G23310	26	101	1.25E-11
AT3G60620	16	81	1.24E-11
AT1G10580	38	123	1.18E-11
AT2G37035	4	52	1.10E-11
AT5G35735	14	77	1.05E-11
AT1G04950	5	55	1.04E-11

AT1G08940	1	42	1.00E-11
AT5G04610	1	42	1.00E-11
AT1G54140	68	173	9.92E-12
AT4G23860	6	58	9.04E-12
AT1G12580	2	46	8.37E-12
AT3G16720	2	46	8.37E-12
AT4G32295	18	86	8.24E-12
AT4G24770	62	164	8.16E-12
AT1G77420	12	73	8.11E-12
AT4G19640	9	66	7.67E-12
AT4G38960	9	66	7.67E-12
AT3G51930	4	53	5.92E-12
AT3G02450	5	56	5.65E-12
AT2G21130	54	152	5.55E-12
AT2G01140	48	142	5.54E-12
AT1G74720	22	95	5.33E-12
AT5G10010	65	170	5.19E-12
AT5G13190	9	67	4.34E-12
AT5G15150	15	81	4.04E-12
AT5G27330	14	79	3.63E-12
AT3G12510	8	65	3.24E-12
AT3G09410	3	51	2.92E-12
AT4G27410	3	51	2.92E-12
AT4G34140	12	75	2.72E-12
AT4G13070	72	183	2.56E-12
AT3G28130	9	68	2.45E-12
AT2G04865	2	48	2.27E-12
AT1G06200	14	80	2.13E-12
AT5G55500	25	103	1.94E-12
AT3G07770	13	78	1.85E-12
AT5G37350	3	52	1.54E-12
AT5G45410	52	152	1.48E-12
AT2G22610	15	83	1.40E-12
AT2G20900	50	149	1.27E-12
AT3G58865	14	81	1.24E-12
AT1G26150	26	106	1.17E-12
AT4G03410	8	67	1.01E-12
AT3G01150	16	86	9.16E-13
AT1G75230	4	56	9.10E-13
AT3G53260	4	56	9.10E-13
AT5G47430	27	109	7.07E-13
AT3G21030	2	50	6.13E-13
AT1G59910	10	73	5.81E-13
AT3G22850	65	176	5.37E-13
AT1G26580	15	85	4.84E-13
AT5G62220	15	85	4.84E-13
AT3G07180	24	104	4.59E-13
AT2G22480	23	102	4.58E-13

AT5G48720	67	180	4.01E-13
AT5G26780	13	81	3.61E-13
AT3G46920	1	47	3.49E-13
AT5G44350	2	51	3.18E-13
AT2G45100	21	99	2.67E-13
AT1G13570	28	113	2.65E-13
AT5G01220	9	72	2.46E-13
AT4G02010	11	77	2.41E-13
AT1G01570	3	55	2.26E-13
AT1G26100	3	55	2.26E-13
AT2G40690	3	55	2.26E-13
AT1G04970	1	48	1.78E-13
AT2G35530	30	118	1.54E-13
AT4G17950	5	62	1.42E-13
AT3G03950	4	59	1.38E-13
AT3G05830	9	73	1.38E-13
AT5G63040	6	65	1.33E-13
AT2G21290	35	128	1.18E-13
AT1G06450	78	201	1.12E-13
AT2G38090	15	88	9.68E-14
AT1G23180	4	60	7.38E-14
AT1G76120	6	66	7.27E-14
AT5G49160	3	57	6.26E-14
AT4G01150	10	77	5.93E-14
AT3G09800	79	205	4.59E-14
AT1G19715	2	54	4.44E-14
AT5G48545	5	64	4.13E-14
AT1G28100	6	67	3.96E-14
AT5G16620	4	61	3.93E-14
AT2G20610	71	193	3.33E-14
AT5G28040	29	120	2.37E-14
AT2G24260	1	51	2.36E-14
AT1G17040	6	68	2.15E-14
AT5G11500	4	62	2.09E-14
AT5G64343	84	216	1.48E-14
AT2G33590	29	121	1.47E-14
AT5G47050	11	82	1.42E-14
AT4G27250	16	94	1.29E-14
AT1G15730	1	52	1.20E-14
AT3G15640	2	56	1.19E-14
AT2G42030	6	69	1.17E-14
AT2G25790	4	63	1.11E-14
AT4G17410	4	63	1.11E-14
AT5G01870	15	92	1.11E-14
AT3G56760	19	101	1.08E-14
AT5G08650	12	85	1.05E-14
AT2G30990	18	99	9.76E-15
AT3G19740	3	60	9.06E-15

AT4G28390	24	112	8.71E-15
AT5G08630	32	128	8.46E-15
AT3G16870	55	170	7.49E-15
AT2G01410	1	53	6.11E-15
AT1G27980	4	64	5.89E-15
AT1G05055	3	61	4.75E-15
AT1G31850	16	96	4.39E-15
AT5G21070	70	197	3.74E-15
AT3G58270	30	126	3.41E-15
AT2G29630	12	87	3.38E-15
AT1G65590	25	116	3.29E-15
AT5G11000	47	158	3.14E-15
AT4G32980	4	65	3.12E-15
AT1G10160	1	54	3.11E-15
AT5G58003	14	92	3.07E-15
AT1G55540	3	62	2.49E-15
AT3G17800	3	62	2.49E-15
AT2G14460	9	80	2.31E-15
AT4G01940	25	117	1.99E-15
AT4G06634	35	137	1.85E-15
AT5G09690	4	66	1.65E-15
AT2G42410	1	55	1.58E-15
AT5G41960	1	55	1.58E-15
AT1G11390	8	78	1.53E-15
AT3G03490	8	78	1.53E-15
AT1G34150	46	159	9.17E-16
AT3G56120	83	222	8.68E-16
AT1G45332	16	99	8.60E-16
AT1G21070	2	60	8.49E-16
AT1G56290	2	60	8.49E-16
AT3G27810	57	179	7.39E-16
AT4G30780	15	97	7.15E-16
AT4G34800	15	97	7.15E-16
AT5G04410	36	141	6.95E-16
AT5G58870	12	90	6.15E-16
AT2G02480	5	71	5.26E-16
AT3G22550	5	71	5.26E-16
AT4G16960	7	77	5.15E-16
AT1G77030	30	130	4.89E-16
AT3G15270	26	122	4.57E-16
AT5G67530	25	120	4.39E-16
AT4G01935	1	57	4.10E-16
AT1G11330	59	184	3.89E-16
AT3G01160	79	218	3.33E-16
AT2G22090	64	193	3.28E-16
AT3G51390	10	86	3.22E-16
AT1G76310	71	205	3.04E-16
AT5G18980	34	139	2.88E-16

AT4G20020	105	260	2.72E-16
AT2G47485	4	69	2.45E-16
AT3G09710	40	151	2.28E-16
AT4G15850	2	62	2.26E-16
AT4G10620	17	104	1.99E-16
AT5G04320	49	168	1.90E-16
AT1G09320	25	122	1.59E-16
AT5G62760	6	76	1.57E-16
AT3G12340	7	79	1.52E-16
AT3G54620	56	181	1.51E-16
AT5G43780	24	120	1.50E-16
AT4G10930	40	152	1.44E-16
AT4G34150	23	118	1.40E-16
AT5G51850	15	100	1.36E-16
AT3G44600	86	232	1.23E-16
AT3G02140	9	85	1.20E-16
AT1G14790	2	63	1.17E-16
AT5G62020	27	127	1.05E-16
AT4G37650	64	196	9.55E-17
AT4G01037	7	80	8.28E-17
AT3G59420	11	91	7.99E-17
AT1G67320	22	117	7.62E-17
AT4G29660	44	161	7.22E-17
AT4G13020	4	71	6.82E-17
AT4G38810	45	163	6.76E-17
AT1G65440	58	187	5.25E-17
AT2G43540	23	120	4.95E-17
AT2G41020	15	102	4.46E-17
AT1G10170	20	114	3.59E-17
AT1G06530	43	161	3.07E-17
AT5G25470	56	185	2.72E-17
AT5G20080	15	103	2.55E-17
AT3G21465	27	130	2.30E-17
AT5G58470	8	85	2.27E-17
AT4G24380	41	158	2.14E-17
AT5G67440	30	137	1.55E-17
AT5G18690	27	131	1.39E-17
AT2G21940	7	83	1.32E-17
AT5G63700	7	83	1.32E-17
AT5G55040	43	163	1.22E-17
AT1G49360	5	77	1.21E-17
AT1G17760	44	165	1.16E-17
AT3G56860	68	208	1.15E-17
AT2G17780	16	107	1.05E-17
AT1G27730	4	74	9.99E-18
AT5G48150	34	146	9.87E-18
AT1G05470	10	92	9.43E-18
AT5G10470	42	162	8.08E-18

AT2G41890	98	259	6.84E-18
AT4G00870	91	248	6.06E-18
AT4G14510	22	122	5.48E-18
AT4G24530	40	159	5.47E-18
AT3G15340	2	68	4.22E-18
AT5G16150	3	72	3.74E-18
AT2G40820	48	175	3.67E-18
AT1G67630	13	102	2.70E-18
AT4G38460	60	198	2.15E-18
AT1G75500	50	180	2.04E-18
AT2G47350	3	73	1.94E-18
AT4G25360	44	169	1.83E-18
AT3G24860	10	95	1.59E-18
AT5G16760	115	290	1.56E-18
AT5G36160	81	235	1.53E-18
AT5G04270	13	103	1.52E-18
AT1G09850	7	87	1.13E-18
AT5G24310	2	70	1.12E-18
AT1G68550	16	111	1.12E-18
AT1G55930	38	159	8.52E-19
AT3G23660	30	143	7.65E-19
AT2G29140	11	99	7.40E-19
AT5G15580	117	296	5.55E-19
AT4G36860	37	158	5.30E-19
AT4G33110	11	100	4.10E-19
AT5G13520	79	236	2.62E-19
AT1G09020	55	194	2.38E-19
AT5G41610	11	101	2.27E-19
AT1G68990	16	114	2.08E-19
AT1G04610	7	90	1.76E-19
AT3G57470	31	148	1.76E-19
AT5G48370	5	84	1.43E-19
AT4G11130	7	91	9.48E-20
AT1G63420	6	88	8.84E-20
AT4G18800	42	172	7.26E-20
AT1G03960	13	109	4.70E-20
AT5G05540	33	155	4.21E-20
AT1G15000	50	189	3.38E-20
AT5G27650	22	132	2.59E-20
AT3G57990	116	303	2.38E-20
AT1G10390	114	300	2.21E-20
AT3G14350	5	87	2.11E-20
AT1G80910	58	205	2.04E-20
AT4G08570	23	135	1.76E-20
AT3G58180	113	299	1.76E-20
AT2G47590	15	116	1.62E-20
AT1G69870	1	72	1.57E-20
AT3G59820	25	140	1.36E-20

AT3G12600	83	251	9.29E-21
AT1G26800	51	194	8.07E-21
AT3G10040	9	101	7.87E-21
AT2G45340	27	146	5.94E-21
AT3G52180	75	239	4.33E-21
AT4G09970	9	102	4.28E-21
AT3G07060	1	74	4.03E-21
AT5G14370	90	265	3.95E-21
AT4G24510	16	121	3.93E-21
AT5G13050	47	188	3.71E-21
AT1G79360	11	108	3.54E-21
AT5G15390	11	108	3.54E-21
AT1G64460	32	158	3.19E-21
AT5G40850	127	326	3.13E-21
AT5G05820	17	124	2.96E-21
AT4G15130	22	136	2.95E-21
AT4G39050	87	261	2.54E-21
AT1G27630	109	298	2.21E-21
AT1G70985	1	75	2.04E-21
AT5G51380	25	144	1.61E-21
AT1G50740	24	142	1.39E-21
AT1G66730	7	99	6.48E-22
AT5G33300	4	89	6.19E-22
AT3G51910	1	77	5.24E-22
AT1G15215	24	144	4.73E-22
AT3G22980	55	208	4.16E-22
AT4G37608	2	82	3.70E-22
AT5G01780	2	82	3.70E-22
AT1G24560	41	181	3.56E-22
AT3G11330	50	199	3.21E-22
AT4G27510	6	97	3.01E-22
AT5G12860	45	190	2.20E-22
AT3G29320	89	271	1.72E-22
AT3G18390	6	98	1.60E-22
AT4G08685	10	111	1.05E-22
AT5G19380	3	88	1.02E-22
AT1G14685	34	170	6.06E-23
AT5G12900	43	189	5.10E-23
AT5G15970	147	370	3.40E-23
AT4G02720	107	307	1.82E-23
AT4G26510	1	82	1.74E-23
AT1G31070	55	215	1.62E-23
AT4G31670	10	115	9.17E-24
AT5G55130	1	83	8.82E-24
AT2G41060	12	121	7.86E-24
AT5G12080	47	201	7.37E-24
AT5G65440	32	170	6.47E-24
AT3G50820	10	116	4.98E-24

AT5G08260	102	302	4.67E-24
AT2G05520	1	84	4.46E-24
AT3G10020	87	277	3.33E-24
AT1G69220	14	128	3.24E-24
AT4G01880	25	156	2.45E-24
AT3G19650	12	123	2.36E-24
AT2G21470	47	204	1.72E-24
AT3G03270	149	382	1.37E-24
AT3G01200	24	155	1.17E-24
AT5G44710	140	369	8.31E-25
AT2G45710	123	342	6.56E-25
AT5G22760	26	161	5.73E-25
AT5G60210	50	214	2.47E-25
AT5G42920	48	210	2.45E-25
AT4G20430	66	246	1.51E-25
AT5G53760	123	346	1.38E-25
AT1G58025	2	94	1.18E-25
AT1G75580	56	228	8.80E-26
AT4G02560	18	145	7.65E-26
AT4G39300	3	99	7.01E-26
AT3G51800	50	217	5.74E-26
AT1G06870	60	237	4.92E-26
AT2G31740	30	175	4.31E-26
AT4G19160	64	245	4.20E-26
AT3G21310	4	104	3.45E-26
AT1G73960	81	277	3.37E-26
AT3G12680	52	223	2.18E-26
AT1G65030	47	213	2.06E-26
AT3G52140	11	128	1.97E-26
AT5G44290	11	128	1.97E-26
AT1G55960	8	119	1.70E-26
AT5G58620	26	168	1.24E-26
AT3G47390	46	212	1.22E-26
AT5G62050	3	102	9.57E-27
AT4G36630	14	138	8.53E-27
AT4G30993	6	114	5.83E-27
AT1G79350	3	103	4.92E-27
AT1G19600	19	153	3.36E-27
AT1G27770	97	311	3.18E-27
AT5G41360	2	100	2.08E-27
AT1G70590	25	170	1.08E-27
AT4G34260	16	147	1.07E-27
AT4G34390	10	130	8.97E-28
AT3G13445	43	211	8.55E-28
AT4G26750	50	226	6.90E-28
AT1G63100	36	197	4.66E-28
AT1G67580	41	208	4.47E-28
AT5G17000	21	162	3.64E-28

AT3G22840	167	434	2.55E-28
AT1G54130	44	216	1.97E-28
AT3G12860	37	201	1.82E-28
AT2G26600	3	108	1.77E-28
AT1G07420	7	123	1.66E-28
AT3G32940	1	99	1.60E-28
AT4G14930	63	255	1.58E-28
AT1G34355	50	230	9.48E-29
AT4G35580	68	266	8.48E-29
AT5G01960	123	365	7.26E-29
AT2G29420	170	443	5.38E-29
AT1G32540	1	101	4.08E-29
AT3G50370	142	399	3.06E-29
AT2G19950	16	154	1.69E-29
AT3G25980	42	217	1.31E-29
AT1G63250	17	158	8.05E-30
AT1G64640	14	150	6.28E-30
AT2G29560	52	240	5.09E-30
AT5G26570	102	335	4.97E-30
AT1G61730	20	167	4.58E-30
AT1G54920	85	305	3.49E-30
AT4G24110	193	488	2.39E-30
AT5G07020	58	254	2.15E-30
AT5G64070	100	334	1.64E-30
AT1G74310	4	120	9.19E-31
AT2G27580	25	183	7.31E-31
AT3G57030	52	244	6.90E-31
AT5G13930	72	285	4.30E-31
AT3G07030	25	184	4.15E-31
AT5G53420	124	380	3.20E-31
AT5G66540	71	284	2.77E-31
AT1G42440	123	379	2.37E-31
AT2G37500	94	329	1.26E-31
AT1G60440	68	281	7.10E-32
AT1G65900	12	152	4.90E-32
AT5G55310	9	143	3.54E-32
AT2G26890	79	304	3.22E-32
AT5G49930	8	140	2.82E-32
AT5G52640	57	261	2.50E-32
AT5G01890	133	402	2.18E-32
AT1G72710	131	401	8.03E-33
AT3G28140	1	114	5.62E-33
AT5G07590	35	216	5.25E-33
AT3G08720	13	161	1.25E-33
AT1G69060	4	130	1.23E-33
AT3G27320	15	167	1.22E-33
AT2G43320	24	193	5.62E-34
AT3G43240	34	219	2.81E-34

AT2G29540	25	197	2.49E-34
AT1G14610	213	545	2.37E-34
AT2G40700	53	262	2.23E-34
AT3G62260	22	191	8.44E-35
AT5G08535	9	153	6.20E-35
AT2G29510	90	340	3.06E-35
ATMG01390	1	122	2.35E-35
AT5G66140	45	250	1.32E-35
AT4G25900	1	123	1.18E-35
AT5G66250	50	262	7.99E-36
AT3G52380	91	345	7.35E-36
AT1G33410	53	269	6.18E-36
AT5G01820	69	303	4.48E-36
AT1G55020	80	325	4.22E-36
AT1G03900	21	194	2.95E-36
AT2G31340	4	140	1.60E-36
AT3G48040	91	349	1.16E-36
AT4G00040	22	200	4.28E-37
AT4G08500	2	133	4.25E-37
AT1G14710	93	355	4.22E-37
AT3G61190	58	286	2.46E-37
AT1G06060	19	193	1.94E-37
AT5G55830	7	155	1.85E-37
AT3G20290	67	307	8.33E-38
AT5G22360	35	237	5.03E-38
AT2G25140	56	285	4.58E-38
AT3G59280	23	207	3.43E-38
AT3G51850	228	594	2.35E-38
AT3G18290	3	142	2.30E-38
AT4G13430	149	464	1.51E-38
AT3G61740	5	153	4.40E-39
AT1G71100	108	394	3.13E-39
AT2G40140	41	257	2.21E-39
AT1G60850	40	255	1.82E-39
AT2G21380	39	253	1.48E-39
AT5G48930	72	327	6.76E-40
AT3G59350	2	143	4.80E-40
AT3G47850	135	449	2.51E-40
AT1G34360	47	275	2.42E-40
AT1G72090	106	396	2.29E-40
AT4G21430	20	210	3.93E-41
AT4G18670	4	156	3.74E-41
AT5G04870	50	288	8.99E-42
AT3G61960	140	466	7.80E-42
AT1G78090	95	384	3.33E-42
AT4G31170	242	645	5.80E-43
AT2G27600	179	542	3.02E-43
AT1G18300	73	350	1.95E-44

AT5G66030	52	307	3.96E-45
AT5G48570	1	157	8.82E-46
AT1G32640	30	256	7.15E-46
AT4G08870	221	631	2.22E-46
AT5G43880	104	422	2.13E-46
AT2G27285	72	358	1.14E-46
AT4G26080	36	275	1.02E-46
AT5G66210	23	240	1.00E-46
AT4G27440	120	455	7.76E-47
AT3G63200	18	227	3.27E-47
AT3G20410	15	219	1.33E-47
AT1G69960	33	272	6.59E-48
AT3G13580	61	341	2.87E-48
AT1G09310	56	330	2.37E-48
AT2G15290	160	539	8.40E-49
AT4G29670	13	223	1.59E-50
AT4G01070	18	240	1.05E-50
AT5G23430	9	210	6.85E-51
AT3G01130	156	545	2.52E-51
AT1G11700	44	314	2.31E-51
AT4G38710	103	445	1.33E-51
AT5G35320	92	424	6.56E-52
AT3G44050	32	288	1.22E-52
AT4G23460	63	365	9.13E-53
AT1G04220	302	804	6.28E-53
AT3G19980	105	457	2.77E-53
AT1G63810	46	329	8.17E-54
AT1G07970	17	252	7.89E-55
AT2G34870	25	278	3.53E-55
AT2G24420	46	339	2.89E-56
AT5G07180	58	370	1.12E-56
AT2G05120	74	407	1.01E-56
AT1G67340	28	294	4.36E-57
AT4G26590	299	829	7.31E-58
AT1G16190	67	398	2.59E-58
AT1G16610	371	950	1.17E-58
AT2G34357	150	574	3.24E-59
AT1G15200	70	409	2.85E-59
AT5G14040	324	880	1.54E-59
AT1G53540	2	209	1.39E-59
AT5G61600	66	402	8.54E-60
AT1G06900	62	399	2.57E-61
AT2G02050	155	601	1.02E-62
AT4G23680	176	643	3.66E-63
AT1G15280	146	598	1.13E-65
AT1G07350	5	246	4.64E-66
AT5G66680	224	752	5.07E-67
AT3G58630	55	411	2.13E-68

AT3G51950	326	942	1.95E-69
AT2G12400	129	582	1.73E-69
AT4G21580	6	263	1.11E-69
AT2G36720	43	388	1.56E-70
AT3G11820	13	297	3.18E-71
AT4G01100	291	893	2.82E-71
AT1G70320	493	1241	4.23E-74
AT3G58780	277	885	3.11E-74
AT5G56950	38	389	2.16E-74
AT3G62330	3	267	3.59E-75
AT3G06450	329	987	2.17E-76
AT5G59390	23	354	2.58E-77
AT1G30590	31	387	2.40E-79
AT3G23820	213	797	1.27E-79
AT2G46240	21	356	1.03E-79
AT3G50070	161	701	6.17E-81
AT4G38620	267	928	1.25E-85
AT2G35060	50	468	4.23E-86
AT2G04880	30	409	4.20E-86
AT3G12050	180	767	8.89E-87
AT1G49890	127	669	1.43E-89
AT3G49530	97	601	1.39E-89
AT4G34410	3	325	2.27E-92
AT2G32120	18	398	2.00E-94
AT3G15095	330	1108	4.07E-98
AT5G09590	98	650	1.11E-100
AT4G30440	123	713	1.09E-101
AT2G01630	0	15	6.10E-05
AT3G53830	0	15	6.10E-05
AT3G55480	0	15	6.10E-05
AT4G08110	0	15	6.10E-05
AT4G19060	0	15	6.10E-05
AT4G26090	0	15	6.10E-05
AT4G36410	0	15	6.10E-05
AT4G38770	0	15	6.10E-05
AT5G06685	0	15	6.10E-05
AT5G44750	0	15	6.10E-05
AT5G63905	0	15	6.10E-05
AT5G65200	0	15	6.10E-05
AT1G68160	0	16	3.05E-05
AT4G28005	0	16	3.05E-05
AT4G28270	0	16	3.05E-05
AT5G22320	0	16	3.05E-05
AT5G12440	3	25	2.74E-05
AT4G25960	12	44	2.09E-05
AT1G26218	0	17	1.53E-05
AT3G01400	0	17	1.53E-05
AT3G05040	0	17	1.53E-05

AT4G21705	0	17	1.53E-05
AT5G56190	0	17	1.53E-05
AT1G20823	2	24	1.05E-05
AT1G08550	3	27	8.43E-06
AT1G10490	0	18	7.63E-06
AT2G30880	0	18	7.63E-06
AT3G26760	0	18	7.63E-06
AT3G51340	0	18	7.63E-06
AT3G61440	0	18	7.63E-06
AT4G33260	0	18	7.63E-06
AT4G39860	0	18	7.63E-06
AT5G01660	0	18	7.63E-06
AT5G44005	0	18	7.63E-06
AT5G47080	0	18	7.63E-06
AT2G28940	1	22	5.72E-06
AT1G09290	0	19	3.82E-06
AT1G16490	0	19	3.82E-06
AT1G31790	0	19	3.82E-06
AT2G25480	0	19	3.82E-06
AT2G35100	0	19	3.82E-06
AT3G07650	0	19	3.82E-06
AT3G10290	0	19	3.82E-06
AT3G11950	0	19	3.82E-06
AT3G16175	0	19	3.82E-06
AT3G21480	0	19	3.82E-06
AT3G52820	0	19	3.82E-06
AT4G19390	0	19	3.82E-06
AT4G25300	0	19	3.82E-06
AT4G33980	0	19	3.82E-06
AT4G36830	0	19	3.82E-06
AT5G06480	0	19	3.82E-06
AT5G09880	0	19	3.82E-06
AT5G19730	0	19	3.82E-06
AT1G06490	0	20	1.91E-06
AT1G22410	0	20	1.91E-06
AT1G52890	0	20	1.91E-06
AT2G20980	0	20	1.91E-06
AT2G31970	0	20	1.91E-06
AT2G36370	0	20	1.91E-06
AT2G42260	0	20	1.91E-06
AT3G13900	0	20	1.91E-06
AT3G46100	0	20	1.91E-06
AT3G49410	0	20	1.91E-06
AT4G27340	0	20	1.91E-06
AT4G33945	0	20	1.91E-06
AT4G39070	0	20	1.91E-06
AT5G01170	0	20	1.91E-06
AT5G38530	0	20	1.91E-06

AT3G14050	12	50	1.22E-06
AT1G57600	0	21	9.54E-07
AT1G76650	0	21	9.54E-07
AT1G78270	0	21	9.54E-07
AT2G11891	0	21	9.54E-07
AT2G27460	0	21	9.54E-07
AT2G33815	0	21	9.54E-07
AT3G17690	0	21	9.54E-07
AT3G18900	0	21	9.54E-07
AT3G23740	0	21	9.54E-07
AT3G23940	0	21	9.54E-07
AT3G26640	0	21	9.54E-07
AT4G28703	0	21	9.54E-07
AT5G52390	0	21	9.54E-07
AT2G32520	1	25	8.05E-07
AT1G20693	0	22	4.77E-07
AT1G26500	0	22	4.77E-07
AT1G67080	0	22	4.77E-07
AT2G28360	0	22	4.77E-07
AT2G33050	0	22	4.77E-07
AT3G09870	0	22	4.77E-07
AT3G14205	0	22	4.77E-07
AT3G21540	0	22	4.77E-07
AT4G12250	0	22	4.77E-07
AT4G23780	0	22	4.77E-07
AT4G34450	0	22	4.77E-07
AT5G25475	0	22	4.77E-07
AT5G26860	0	22	4.77E-07
ATCG01100	0	22	4.77E-07
AT2G32170	0	23	2.38E-07
AT2G37310	0	23	2.38E-07
AT3G57350	0	23	2.38E-07
AT4G28570	0	23	2.38E-07
AT5G23530	0	23	2.38E-07
AT5G25610	0	23	2.38E-07
AT5G27930	0	23	2.38E-07
AT5G47445	0	23	2.38E-07
AT5G49720	0	23	2.38E-07
AT5G61390	0	23	2.38E-07
ATMG01360	0	23	2.38E-07
AT1G26530	3	34	1.23E-07
AT1G62640	0	24	1.19E-07
AT2G03280	0	24	1.19E-07
AT3G02010	0	24	1.19E-07
AT3G15580	0	24	1.19E-07
AT3G15870	0	24	1.19E-07
AT4G08470	0	24	1.19E-07
AT4G19130	0	24	1.19E-07

AT4G23540	0	24	1.19E-07
AT4G35000	0	24	1.19E-07
AT5G14120	0	24	1.19E-07
AT5G26870	0	24	1.19E-07
AT5G46540	0	24	1.19E-07
AT5G64760	0	24	1.19E-07
AT3G05580	1	28	1.12E-07
AT1G77885	0	25	5.96E-08
AT2G36460	0	25	5.96E-08
AT2G40400	0	25	5.96E-08
AT5G16790	0	25	5.96E-08
AT5G61300	0	25	5.96E-08
AT1G09155	0	26	2.98E-08
AT1G15510	0	26	2.98E-08
AT1G67190	0	26	2.98E-08
AT1G69030	0	26	2.98E-08
AT2G40230	0	26	2.98E-08
AT2G40520	0	26	2.98E-08
AT2G40850	0	26	2.98E-08
AT2G45400	0	26	2.98E-08
AT3G03140	0	26	2.98E-08
AT3G27770	0	26	2.98E-08
AT4G23250	0	26	2.98E-08
AT4G39795	0	26	2.98E-08
AT5G04430	0	26	2.98E-08
AT5G21960	0	26	2.98E-08
AT5G60450	0	26	2.98E-08
AT5G63770	0	26	2.98E-08
AT5G63890	0	26	2.98E-08
AT5G64820	0	26	2.98E-08
AT2G20830	2	34	1.94E-08
AT1G30130	0	27	1.49E-08
AT2G17370	0	27	1.49E-08
AT2G22660	0	27	1.49E-08
AT3G02260	0	27	1.49E-08
AT3G18060	0	27	1.49E-08
AT3G49510	0	27	1.49E-08
AT4G06676	0	27	1.49E-08
AT4G09012	0	27	1.49E-08
AT4G15140	0	27	1.49E-08
AT4G27650	0	27	1.49E-08
AT5G10840	0	27	1.49E-08
AT5G38640	0	27	1.49E-08
AT5G56040	0	27	1.49E-08
AT1G03530	0	28	7.45E-09
AT1G05135	0	28	7.45E-09
AT1G68680	0	28	7.45E-09
AT1G70505	0	28	7.45E-09

AT2G23980	0	28	7.45E-09
AT2G36590	0	28	7.45E-09
AT2G45460	0	28	7.45E-09
AT3G03470	0	28	7.45E-09
AT4G14580	0	28	7.45E-09
AT4G21490	0	28	7.45E-09
AT4G25440	0	28	7.45E-09
AT4G39570	0	28	7.45E-09
AT5G03720	0	28	7.45E-09
AT5G19940	0	28	7.45E-09
AT5G20160	0	28	7.45E-09
AT5G35560	0	28	7.45E-09
AT5G54630	0	28	7.45E-09
AT5G56240	2	36	5.40E-09
AT3G12580	1	33	4.08E-09
AT1G14740	0	29	3.73E-09
AT1G26570	0	29	3.73E-09
AT1G45050	0	29	3.73E-09
AT1G53110	0	29	3.73E-09
AT2G25470	0	29	3.73E-09
AT2G31060	0	29	3.73E-09
AT3G49630	0	29	3.73E-09
AT3G59550	0	29	3.73E-09
AT5G02750	0	29	3.73E-09
AT5G06290	0	29	3.73E-09
AT1G03080	0	30	1.86E-09
AT1G59600	0	30	1.86E-09
AT2G21770	0	30	1.86E-09
AT4G12000	0	30	1.86E-09
AT4G17750	0	30	1.86E-09
AT4G34000	0	30	1.86E-09
AT5G15490	0	30	1.86E-09
AT5G25280	0	30	1.86E-09
AT5G41140	0	30	1.86E-09
AT1G14430	0	31	9.32E-10
AT1G69180	0	31	9.32E-10
AT3G06270	0	31	9.32E-10
AT3G49750	0	31	9.32E-10
AT5G24160	0	31	9.32E-10
AT5G36220	0	31	9.32E-10
AT5G49940	0	31	9.32E-10
AT5G50240	0	31	9.32E-10
AT1G69780	2	39	7.85E-10
AT1G26340	0	32	4.66E-10
AT1G80160	0	32	4.66E-10
AT2G19450	0	32	4.66E-10
AT3G44326	0	32	4.66E-10
AT3G49900	0	32	4.66E-10

AT5G16730	0	32	4.66E-10
AT5G38690	0	32	4.66E-10
AT5G47420	0	32	4.66E-10
AT1G50040	0	33	2.33E-10
AT1G70740	0	33	2.33E-10
AT4G01090	0	33	2.33E-10
AT4G21310	0	33	2.33E-10
AT5G18620	0	33	2.33E-10
AT3G51250	1	38	1.46E-10
AT3G50900	0	34	1.16E-10
AT3G55730	0	34	1.16E-10
AT3G56810	0	34	1.16E-10
AT5G19450	0	34	1.16E-10
AT5G46390	0	34	1.16E-10
AT3G13225	1	39	7.46E-11
AT1G07985	0	35	5.82E-11
AT1G54610	0	35	5.82E-11
AT1G62310	0	35	5.82E-11
AT2G22190	0	35	5.82E-11
AT3G18610	0	35	5.82E-11
AT1G63700	0	36	2.91E-11
AT3G06210	0	36	2.91E-11
AT3G61780	0	36	2.91E-11
AT4G24900	0	36	2.91E-11
AT4G31500	0	36	2.91E-11
AT4G32840	0	36	2.91E-11
AT5G08390	2	45	1.61E-11
AT1G63940	0	37	1.46E-11
AT1G76110	0	37	1.46E-11
AT1G78950	0	37	1.46E-11
AT3G07760	0	37	1.46E-11
AT3G16785	0	37	1.46E-11
AT3G29150	0	37	1.46E-11
AT4G25490	0	37	1.46E-11
ATMG00040	0	37	1.46E-11
AT1G13030	0	38	7.28E-12
AT2G44200	0	38	7.28E-12
AT3G12400	0	38	7.28E-12
AT3G21010	0	38	7.28E-12
AT4G00990	0	38	7.28E-12
AT4G08700	0	38	7.28E-12
AT5G17250	0	38	7.28E-12
AT4G18120	2	47	4.36E-12
AT1G02290	0	39	3.64E-12
AT1G57820	0	39	3.64E-12
AT2G32250	0	39	3.64E-12
AT2G43430	0	39	3.64E-12
AT2G44940	0	39	3.64E-12

AT3G11650	0	39	3.64E-12
AT4G12680	0	39	3.64E-12
AT4G18160	0	39	3.64E-12
AT1G49720	0	40	1.82E-12
AT1G51630	0	40	1.82E-12
AT3G55560	0	40	1.82E-12
AT4G10550	0	40	1.82E-12
AT2G01460	0	41	9.10E-13
AT3G05880	0	41	9.10E-13
AT3G18640	0	41	9.10E-13
AT4G14965	0	41	9.10E-13
AT4G19590	0	41	9.10E-13
AT1G02070	0	42	4.55E-13
AT1G04980	0	42	4.55E-13
AT1G72520	0	42	4.55E-13
AT3G02910	0	42	4.55E-13
AT5G57080	0	42	4.55E-13
AT5G62430	0	42	4.55E-13
AT3G09840	0	43	2.28E-13
AT3G56790	0	43	2.28E-13
AT4G24390	0	43	2.28E-13
AT1G76260	0	44	1.14E-13
AT2G45300	0	44	1.14E-13
AT5G05130	0	44	1.14E-13
AT1G16800	0	45	5.69E-14
AT1G19180	0	45	5.69E-14
AT3G21600	0	45	5.69E-14
AT4G10690	0	45	5.69E-14
AT5G52830	0	45	5.69E-14
AT1G21050	0	46	2.85E-14
AT1G21350	0	46	2.85E-14
AT2G13370	0	46	2.85E-14
AT3G63330	0	46	2.85E-14
AT5G63200	0	46	2.85E-14
AT1G64570	0	47	1.42E-14
AT3G01040	0	47	1.42E-14
AT3G01850	0	47	1.42E-14
AT3G25495	0	47	1.42E-14
AT4G33520	0	47	1.42E-14
AT3G46780	0	48	7.11E-15
AT3G59880	0	48	7.11E-15
AT5G22050	0	48	7.11E-15
AT1G16030	0	49	3.56E-15
AT2G43010	0	49	3.56E-15
AT3G44940	0	49	3.56E-15
AT5G27460	0	49	3.56E-15
AT1G36990	0	50	1.78E-15
AT2G25570	0	50	1.78E-15

AT2G41960	0	50	1.78E-15
AT5G05730	0	50	1.78E-15
AT5G57980	0	50	1.78E-15
AT1G59830	0	51	8.89E-16
AT2G28760	0	51	8.89E-16
AT3G54020	0	51	8.89E-16
AT4G22320	0	51	8.89E-16
AT5G57840	0	51	8.89E-16
AT1G74250	0	52	4.45E-16
AT3G10060	0	52	4.45E-16
AT3G07690	1	57	4.10E-16
AT5G15880	0	53	2.22E-16
AT1G14870	0	54	1.11E-16
AT5G40830	0	54	1.11E-16
AT3G01490	0	55	5.56E-17
AT5G43500	0	55	5.56E-17
AT5G62210	0	55	5.56E-17
AT4G36690	0	56	2.78E-17
AT1G04620	0	57	1.39E-17
AT1G50660	0	57	1.39E-17
AT1G52560	0	57	1.39E-17
AT1G66340	0	57	1.39E-17
AT2G04520	0	57	1.39E-17
AT3G25410	0	58	6.95E-18
AT1G22490	0	59	3.48E-18
AT4G10430	0	59	3.48E-18
AT1G53680	0	60	1.74E-18
AT5G56730	0	60	1.74E-18
AT1G71420	0	61	8.69E-19
AT2G45950	0	61	8.69E-19
AT3G04730	0	61	8.69E-19
AT4G25030	0	61	8.69E-19
AT1G06240	0	62	4.35E-19
AT1G06740	0	62	4.35E-19
AT3G55080	0	62	4.35E-19
AT1G14010	0	63	2.17E-19
AT4G33790	0	63	2.17E-19
AT1G12210	0	64	1.09E-19
AT3G60260	0	64	1.09E-19
AT1G51370	0	65	5.43E-20
AT2G17440	0	65	5.43E-20
AT5G63990	0	65	5.43E-20
AT3G17090	0	66	2.72E-20
AT1G16900	0	67	1.36E-20
AT2G20850	0	67	1.36E-20
AT3G11880	0	68	6.79E-21
AT3G17365	0	68	6.79E-21
AT1G16540	0	69	3.40E-21

AT1G49840	0	70	1.70E-21
AT5G24630	0	70	1.70E-21
AT5G46180	0	70	1.70E-21
AT3G19515	0	71	8.49E-22
AT3G03210	0	73	2.12E-22
AT5G52510	0	73	2.12E-22
AT4G37400	0	74	1.06E-22
AT5G12020	0	78	6.64E-24
AT5G66470	0	78	6.64E-24
AT3G50700	0	79	3.32E-24
AT4G03000	0	79	3.32E-24
AT1G31770	0	80	1.66E-24
AT2G18630	0	84	1.04E-25
AT5G60490	0	84	1.04E-25
AT1G47610	0	85	5.19E-26
AT5G55920	0	85	5.19E-26
AT5G64860	0	86	2.60E-26
AT5G50840	0	87	1.30E-26
AT5G64510	0	88	6.49E-27
AT2G35940	0	93	2.03E-28
AT5G41760	0	93	2.03E-28
AT4G16190	0	94	1.01E-28
AT5G47040	0	96	2.54E-29
AT1G30000	0	97	1.27E-29
AT5G11980	0	107	1.24E-32
AT1G05950	0	110	1.55E-33
AT3G07100	0	121	7.58E-37
AT4G25200	0	131	7.41E-40
AT5G55250	0	135	4.64E-41
AT2G46960	0	149	2.84E-45
AT1G11210	0	170	1.36E-51
AT3G25840	0	187	1.04E-56
AT5G52180	0	209	2.49E-63
AT1G54020	0	299	2.06E-90
AT3G51780	0	353	1.16E-106
AT1G07400	0	604	3.59E-182
