# Supplementary material for: Functional Analysis of Sporophytic Transcripts Repressed by the Female Gametophyte in the Ovule of Arabidopsis thaliana
Source: PLoS One. 2013 Oct 23;8(10):e76977. doi: 10.1371/journal.pone.0076977 (PMC3806734; doi:10.1371/journal.pone.0076977)
Supplement: Table S2 — Pfam analysis of 1335 upregulated genes in spl ovules. (PDF) [file pone.0076977.s006.pdf]

Table S2. Pfam analysis of 1335 upregulated genes in *spl* ovules.

Class name	Number of genes (%)	Description [a]
Signalling	149(9.8%)	Kinase and leucine-rich repeat domain, calmodulins, armadillo proteins, hormone related and stress-induced.
Cell cycle	25(1.7%)	Proteins involved in the mitotic cell cycle.
Organelle processes	69(4.5%)	Proteins involved in organelle activity, mainly chloroplast and mitochondria.
RNA binding	116(7.6%)	RNA binding domain proteins; RNA helicases, Pumilio proteins, RNA methytransferases, ribosomal proteins and RNA capping enzymes.
DNA binding	66(4.4%)	Proteins containing DNA binding domains that are not transcription factors like gyrases, topoisomerases, etc.
Transcription factors	89(5.9%)	Transcription factors
Chromatin related	32(2.1%)	Histone and histone interacting proteins.
Housekeeping processes	286(18.9%)	Proteins involved in cellular trafficking, ubiquitination, photosynthesis, multimerization, chaperones.
Metabolism	354(23.3%)	Epimerases, transferases, hydrolases, lipases, AMP binding proteins, peptidases, etc.
Others	149(9.8%)	Include all genes that could not be classified in other category, mostly those with DUF domains.
Multiple function	16(1%)	Include those genes with multiple domains and multiple functions.
No Pfam Domains	166(11%)	Include all genes without Pfam domain like unknown proteins, TE elements and non-coding RNAs.
Total genes	1517	

[a] Description is based on Pfam domains
