# Supplementary material for: Functional Analysis of Sporophytic Transcripts Repressed by the Female Gametophyte in the Ovule of Arabidopsis thaliana
Source: PLoS One. 2013 Oct 23;8(10):e76977. doi: 10.1371/journal.pone.0076977 (PMC3806734; doi:10.1371/journal.pone.0076977)
Supplement: Table S3 — Phenotypic quantification in At1g47610 and At2g46680 transformed lines using the CaMV35S, pNUC or pES1 promoter. (PDF) [file pone.0076977.s007.pdf]

Table S3. Phenotypic quantification in At1g47610 and At2g46680 transformed lines using the CaMV35S, pNUC or pES1 promoter.

Transgenic line	Number of independent T1 lines	Phenotypes associated with reduced fertility				
		aborted ovules				aborted seeds
		>55%	40-55%	40-20%	<20%	
CaMV35S:AT1G47610	10	1	2	2	0	0
pNUC:At1g47610	21	2	1	1	0	0
pES1:At1g47610	16	0	0	0	0	0
CaMV35S:At2g46680	10	1	2	0	0	0
pNUC:At2g46680	11	1	1	0	0	0
pES1:At2g46680	21	0	2	2	1	1

All show significantly reduced fertility as compared to the wild type;X^2obs.>$X^2$0.05[1]=3.84
