# Supplementary material for: Functional Analysis of Sporophytic Transcripts Repressed by the Female Gametophyte in the Ovule of Arabidopsis thaliana
Source: PLoS One. 2013 Oct 23;8(10):e76977. doi: 10.1371/journal.pone.0076977 (PMC3806734; doi:10.1371/journal.pone.0076977)
Supplement: Table S4 — Quantification of reduced fertility in At1g47610 and At2g46680 selected transformant lines. (PDF) [file pone.0076977.s008.pdf]

Table S4. Quantification of reduced fertility in At1g47610 and At2g46680 selected transformant lines.

Transgenic line T1	Viable seeds	Aborted ovules	Aborted seeds	T2	
				Viable seeds	Aborted ovules
CaMV35S:AT1G47610-2	221(68.4)	89(27.5)	13(4)	ND	ND
CaMV35S:AT1G47610-3	76(40.4)	112(59.6)	0	461(67.5)	222(32.5)
CaMV35S:AT1G47610-6	144(45.7)	171(54.3)	0	130(31.2)	287(68.8)
CaMV35S:AT1G47610-9	88(60.2)	48(32.8)	10(6.8)	ND	ND
CaMV35S:AT1G47610-10	87(57.6)	64(42.4)	0	ND	ND
pNUC:At1g47610-1	192(69.5)	80(29)	4(1.5)	ND	ND
pNUC:At1g47610-6	124(40.8)	175(57.6)	5(1.6)	386(41)	555(59)
pNUC:At1g47610-14	76(25.8)	218(74.1)	0	204(25)	612(75)
pNUC:At1g47610-17	143(45.7)	170(54.3)	0	324(46)	381(54)
CaMV35S:At2g46680-4	68(43.9)	87(56.1)	0	377(51.6)	354(48.4)
CaMV35S:At2g46680-7	76(48.4)	81(51.6)	0	497(54.6)	413(45.4)
CaMV35S:At2g46680-8	78(54.9)	64(45)	0	686(70.5)	287(29.5)
pNUC:At2g46680-1	100(64.9)	54(35.1)	0	683(66.4)	346(33.6)
pNUC:At2g46680-9	39(35.1)	72(64.9)	0	355(30.2)	819(69.8)
pES1:At2g46680-1	77(55.8)	61(44.2)	0	ND	ND
pES1:At2g46680-2	104(74.2)	36(26.8)	0	ND	ND
pES1:At2g46680-9	113(80.8)	27(19.2)	0	ND	ND
pES1:At2g46680-12	134(74)	9(4.9)	38(21)	ND	ND
pES1:At2g46680-18	90(58)	65(42)	0	ND	ND
pES1:At2g46680-23	104(63)	61(37)	0	ND	ND

All show significantly reduced fertility as compared to the wild type;X^2obs.>$X^2$0.05[1]=3.84
