# Supplementary material for: Functional Analysis of Sporophytic Transcripts Repressed by the Female Gametophyte in the Ovule of Arabidopsis thaliana
Source: PLoS One. 2013 Oct 23;8(10):e76977. doi: 10.1371/journal.pone.0076977 (PMC3806734; doi:10.1371/journal.pone.0076977)
Supplement: Table S5 — Gene expression comparison between MPSS and ATH1 microarray results. (PDF) [file pone.0076977.s009.pdf]

Table S5. Gene expression comparison between MPSS and ATH1 microarray results.

Accession	Gene Description	MPSS			ATH1[a]		
		wt	*spl*	fold	wt	mutant	fold
**AT5G53830**	VQ motif-containing protein	2	17	8.50	8.49	30.75	3.62
**AT4G05040**	ankyrin repeat family protein	1	16	16.00	4.45	14.17	3.18
**AT3G52770**	LITTLE ZIPPER 3, ZPR3	2	33	16.50	46.53	119.45	2.57
**AT1G22710**	ARABIDOPSIS THALIANA SUCROSE-PROTON SYMPORTER 2, ATSUC2, SUC2	23	94	4.09	46.59	105.25	2.26
**AT3G28130**	nodulin MtN21 /EamA-like transporter family protein	9	68	7.56	140.76	378.39	2.69
**AT3G22550**	Protein of unknown function (DUF581)	5	71	14.20	16.59	49.15	2.96
**AT4G38810**	Calcium-binding EF-hand family protein;	45	163	3.62	37.57	92.30	2.06
**AT4G08685**	Encodes a protein, expressed in leaves, with similarity to pollen allergens.	10	111	11.10	259.77	696.00	2.20
**AT1G75580**	SAUR-like auxin-responsive protein family	56	228	4.07	271.40	565.13	2.17
**AT3G22840**	EARLY LIGHT-INDUCABLE PROTEIN, ELIP, ELIP1	167	434	2.60	323.93	1164.00	2.89
**AT1G32540**	Encodes a protein with 3 plant-specific zinc finger domains that acts as a positive regulator of cell death.	1	101	101.00	48.03	175.70	3.01
**AT1G61730**	DNA-binding storekeeper protein-related transcriptional regulator;	20	167	8.35	419.87	1510.00	3.75
**AT5G08535**	D111/G-patch domain-containing protein	9	153	17.00	173.67	344.33	2.06
**AT2G34870**	MATERNAL EFFECT EMBRYO ARREST 26, MEE26	25	278	11.12	167.10	586.97	2.87

**AT4G23680**	Polyketide cyclase/dehydrase and lipid transport superfamily protein	176	643	3.65	78.43	681.93	7.13
**AT2G35060**	K+ UPTAKE PERMEASE 11, KUP11	50	468	9.36	47.23	122.77	2.12
**AT5G52390**	PAR1 protein	0	21	21.00	36.36	76.66	2.11
**AT2G36590**	ATPROT3, PROLINE TRANSPORTER 3, PROT3	0	28	28.00	19.05	74.33	3.90
**AT5G41140**	Myosin heavy chain-related protein;	0	30	30.00	8.27	32.14	3.89
**AT1G02070**	unknown protein;	0	42	42.00	68.37	212.43	2.57
**AT1G14870**	ATPCR2, PCR2, PLANT CADMIUM RESISTANCE 2	0	54	54.00	23.03	76.80	2.75
**AT5G62210**	Embryo-specific protein 3, (ATS3);	0	55	55.00	56.23	194.20	2.70
**AT3G19515**	Apoptosis inhibitory 5	0	71	71.00	131.30	530.73	4.22
**AT1G47610**	Transducin/WD40 repeat-like superfamily protein	0	85	85.00	88.67	261.23	2.43

a) Results reported in Johnston et al (2007)
