# Supplementary material for: Functional Analysis of Sporophytic Transcripts Repressed by the Female Gametophyte in the Ovule of Arabidopsis thaliana
Source: PLoS One. 2013 Oct 23;8(10):e76977. doi: 10.1371/journal.pone.0076977 (PMC3806734; doi:10.1371/journal.pone.0076977)
Supplement: Table S6 — Primers used to analyze expression of candidate genes in spl and wild-type ovules. (PDF) [file pone.0076977.s010.pdf]

Table S6. Primers used to analyze expression of candidate genes in *spl* and wild-type ovules.

Gene	Primer	Sequence	L
**At2g25790**	F-At2g25790	ACGGAAGTTTGGTCAAGTGG	20
	R-At2g25790	ATGGCCGTACATTTCAAAGC	20
**At3g24500**	F-At3g24500	TTCGCGTTGTTCCTTTCTCT	20
	R-At3g24500	CCGGCATCGAATTTCTTAAC	20
**At2g35940**	F-At2g35940	GGATTTCAGCTCCAACGAGA	20
	R-At2g35940	GGTCTGCATGAAACCCTGAT	20
**At1g47610**	F-At1g47610	GTGCGGCGGATAAGAAGATA	20
	R-At1g47610	ACCACCGCCAAACACTTAAC	20
**At4g10850**	F-At4g10850	TTGGTTTGGGTTCTTTACGG	20
	R-At4g10850	CGTTGAAAACGCAACATACG	20
**At5g52390**	F-At5g52390	GACGATGTGAAGTGCGAGAA	20
	R-At5g52390	CAACAATGCGTCCGAAGATA	20
**At1g02070**	F-At1g02070	GCTAGACATGGCGGTTCTTC	20
	R-At1g02070	CCGTCTTAGGCGACGTAACT	20
**At2g46680**	F-At2g46680	GACCGGAGATGGAGATGAAA	20
	R-At2g46680	CTTCCTCAAACCCACCAAAA	20
**At4g16190**	F-At4g16190	GCCGCAAGTTTTTAGGACTG	20
	R-At4g16190	AATGACCAGCATGAACCACA	20
**At5g60490**	F-At5g60490	TCGTTAACCGATGAGCAACA	20
	R-At5g60490	GTTACGCCGGACGTGATATT	20
**At1g76110**	F-At1g76110	AATCCCTCAACGAGCAAAGA	20
	R-At1g76110	TGGGCCGAATGATAAAGAAC	20
